# Lipolysis supports bone formation by providing osteoblasts with endogenous fatty acid substrates to maintain bioenergetic status

**DOI:** 10.1038/s41413-023-00297-2

**Published:** 2023-11-24

**Authors:** Ananya Nandy, Ron C. M. Helderman, Santosh Thapa, Shobana Jayapalan, Alison Richards, Nikita Narayani, Michael P. Czech, Clifford J. Rosen, Elizabeth Rendina-Ruedy

**Affiliations:** 1 Department of Medicine, Division of Clinical Pharmacology, Vanderbilt University Medical Center, Nashville, TN 37232 USA; 2 Program in Molecular Medicine, University of Massachusetts Chan Medical School, Worcester, MA 01605 USA; 3 Maine Medical Center Research Institute, Scarborough, ME USA; 4 Molecular Physiology and Biophysics, Vanderbilt University, Nashville, TN 37232 USA

**Keywords:** Bone, Fat metabolism

## Abstract

Bone formation is a highly energy-demanding process that can be impacted by metabolic disorders. Glucose has been considered the principal substrate for osteoblasts, although fatty acids are also important for osteoblast function. Here, we report that osteoblasts can derive energy from endogenous fatty acids stored in lipid droplets via lipolysis and that this process is critical for bone formation. As such, we demonstrate that osteoblasts accumulate lipid droplets that are highly dynamic and provide the molecular mechanism by which they serve as a fuel source for energy generation during osteoblast maturation. Inhibiting cytoplasmic lipolysis leads to both an increase in lipid droplet size in osteoblasts and an impairment in osteoblast function. The fatty acids released by lipolysis from these lipid droplets become critical for cellular energy production as cellular energetics shifts towards oxidative phosphorylation during nutrient-depleted conditions. In vivo, conditional deletion of the ATGL-encoding gene *Pnpla2* in osteoblast progenitor cells reduces cortical and trabecular bone parameters and alters skeletal lipid metabolism. Collectively, our data demonstrate that osteoblasts store fatty acids in the form of lipid droplets, which are released via lipolysis to support cellular bioenergetic status when nutrients are limited. Perturbations in this process result in impairment of bone formation, specifically reducing ATP production and overall osteoblast function.

## Introduction

The skeleton serves a number of physiological functions in mammals by supporting movement, providing protection to internal soft tissues, and generating a reservoir for calcium and phosphorous.^1^ In this regard, bone is constantly functioning to maintain homeostasis between two energy consuming processes: the removal of old bone via bone resorption by the osteoclast and the subsequent formation of new bone by the osteoblast.^2,3^ This equilibrium is maintained by coordinated action involving a complex series of coupled processes and communication between these primary bone cells as well as contributions from the mechanosensing osteocytes.^4^ The formation of new bone requires the differentiation of mesenchymal or stromal cells into ‘mature’ matrix-secreting osteoblasts capable of synthesizing extracellular matrix (ECM) and mineralization vesicles.^5^ These processes within osteoblasts require cellular energy generated from adenosine triphosphate (ATP).^6–8^ In addition to these osteoblast-specific ATP demands, more general cellular processes have been demonstrated to require ATP for chromatin remodeling, active transport, and lysosomal functioning, all of which can impact osteoblast activity.^9–11^ Therefore, osteoblast ATP production or cellular bioenergetic status can directly impact bone formation and overall skeletal health; however, some of these molecular mechanisms orchestrating such processes remain elusive. In this regard, cellular functions of bone formation will continue if, *and only if*, there is sufficient energy to power them.

ATP is derived from metabolic processes utilizing substrates including glucose, amino acids, and fatty acids. Initial studies have described glucose utilization via aerobic glycolysis^12–14^ as integral for osteoblast differentiation and function. Nonetheless, while these cells demonstrate a glycolytic phenotype, they also rely on oxidative metabolism for ATP generation. Osteoblasts have been shown to utilize energy generated by fatty acid oxidation. In this capacity, fatty acid catabolism provides more energy per molecule compared to glucose, and it has been estimated that fatty acids can provide as much as 40%-80% of the energy generated in osteoblasts.^15^ More recently, elegant studies have confirmed the importance of fatty acid mitochondrial oxidation by demonstrating that blocking this process through osteoblast-specific knockdown of an obligatory enzyme in this pathway, *Cpt2*, results in a low-bone-mass phenotype.^16^ Taken together, these data emphasize the ability of osteoblasts to utilize energy-dense fatty acid substrates to presumably generate ATP and support bone formation.

While these data provide evidence that fatty acid catabolism is essential for proper osteoblast function, the origin or source of fatty acid substrates has not been clarified. To this point, our previous work showed that the progression of ex vivo osteoblastogenesis is associated with the accumulation of intracellular lipid droplets in matrix-secreting osteoblasts.^17^ Furthermore, inhibition of lipid droplet synthesis impairs osteoblast differentiation and function, suggesting that these droplets may be a reservoir important for differentiation; however, the molecular mechanism remains unclear.^17^ Given the ATP demand for osteoblast function, we hypothesize that these fatty acids must first be liberated via ATGL-mediated lipolysis to provide those energy-rich substrates. To investigate this, we employed multiple approaches to demonstrate that cytosolic lipolysis supports bone formation by altering the bioenergetic status or ATP generation of osteoblasts. We show that lipid droplets provide free fatty acid substrates for mitochondrial oxidation to generate ATP, enabling proper osteoblast function. This process is particularly physiologically relevant when exogenous nutrient substrates are scarce.

## Results

### Ex vivo differentiation of stromal cells to osteoblasts is associated with altered lipid metabolism

**Fig. 1:**
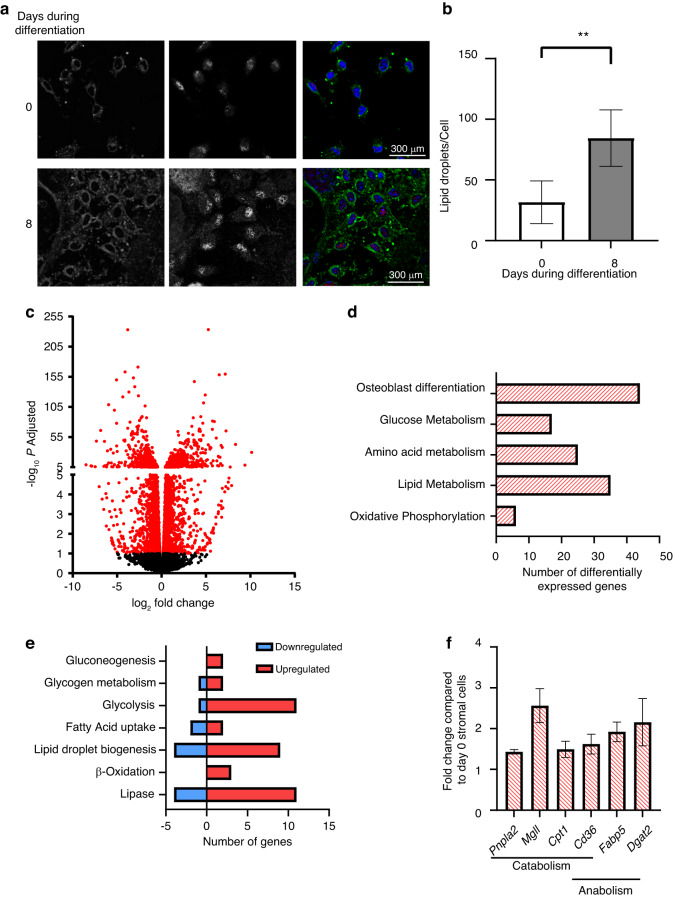
Lipid metabolism during osteoblastogenesis. **a** Representative confocal image of undifferentiated stromal cells on the 0^th^ day and differentiated osteoblasts on the 8^th^ day of ex vivo differentiation from mice. Cells were immunostained for Runx2 (red in merged panel) and mounted with DAPI (blue in merged panel). Cellular lipid droplets were stained with BODIPY 493/503 (green in merged panel). Panels 1 and 2 show monochrome images of lipid droplets and Runx2-stained nuclei, respectively, whereas Panel 3 shows a merged image. **b** Quantification of the number of lipid droplets per cell in stromal cells (open) and differentiated osteoblasts on the 8^th^ day of differentiation (gray). The data are representative of 3 independent experiments (*n* = 3) and are the mean ± standard deviation (SD), where the lipid droplets per cell were counted from independent images captured in 6 different fields of view (*n* = 6) in each experiment. The number of lipid droplets counted from each image was divided by the number of cells (number of DAPI-positive nuclei) in that image to obtain lipid droplets per cell. *t* tests or nonparametric Mann‒Whitney tests were performed accordingly after testing of the normal distribution using the Shapiro‒Wilk normality test to determine significance between two groups where *P < 0.05, **P < 0.01, ***P < 0.001, ****P < 0.000 1. **c** Volcano plot showing differentially expressed genes (FDR-adjusted *P* < 0.05 in red circles) in stromal cells versus differentiated osteoblasts in pairwise comparisons. **d** Distribution of differentially expressed genes belonging to bone formation or metabolic pathways. **e** Distribution of differentially expressed genes belonging to specific glucose or lipid metabolism pathways. **f** Fold changes in the expression of genes involved in lipid metabolism that were statistically significant

To determine how lipid metabolism is altered throughout osteogenic differentiation, bone marrow stromal cells (BMSCs) were stained with BODIPY493/503 prior to osteogenic differentiation, Day 0, or as mature, matrix-secreting osteoblasts following 7 days in osteogenic medium (8^th^ day of differentiation). Undifferentiated stromal cells showed minimal amounts of neutral lipid stored as lipid droplets, as indicated by BODIPY 493/503 staining (Fig. 1a). As our laboratory has previously reported,^17^ we observed accumulation of lipid droplets in mature RUNX2-positive osteoblasts compared to undifferentiated stromal cells (Fig. 1a). Further, quantification showed that undifferentiated stromal cells had 31.67 ± 17.56 lipid droplets per cell, while mature osteoblasts had 84.45 ± 23.25 droplets per cell (Fig. 1b). We confirmed that the lipid accumulation was not due to merely keeping the stromal cells in culture for 7 days, as they were only present under osteogenic conditions (Fig. S1a). We further confirmed that this phenomenon occurred in osteoblasts from other sources, as lipid droplets were detected in mature osteoblasts harvested from the calvarium (Fig. S1b). To further characterize the overall metabolic profile during osteoblastogenesis, bulk RNA sequencing was performed. Data were subjected to analysis for differentially expressed genes (at FDR adjusted *P* value/q value ≤ 0.05). We combined data from 3 replicates from differentiated osteoblasts that were cultured in osteogenic medium for 7 days and 2 replicates from undifferentiated stromal cells to obtain an average of 450 million and 410 million reads, respectively (Table S1). Of the differentially expressed genes between the two groups, 1 239 genes were found to be upregulated and 1 255 genes were downregulated in differentiated osteoblasts compared to stromal cells with a cutoff of 1.5-fold (Fig. 1c and Table S2). Differential expression of 43 genes involved in osteoblastogenesis confirmed the differentiation of stromal cells to osteoblasts after 7 days in osteogenic medium (Fig. 1d). Given the focus on metabolic processes during osteoblastogenesis, significantly differentially expressed genes involved in various metabolic pathways were categorized based on their functions (Fig. 1d). We found that 17 genes involved in glucose metabolism, 25 in amino acid metabolism, 35 genes in lipid metabolism, and 6 genes from the oxidative phosphorylation pathway were differentially expressed (Fig. 1d and Table S2). Further detailed analysis revealed that out of the 17 differentially expressed genes belonging to the glucose metabolism category, 2 gluconeogenesis genes, 2 glycogen metabolism genes, and 11 glycolysis genes were upregulated, whereas 1 glycolysis gene and 1 glycogen metabolism regulatory gene were downregulated (Fig. 1e and Table S2). The only gene downregulated from the glycolysis pathway was *Ldhb*. Among the 35 differentially expressed genes from lipid metabolism, 11 lipases were upregulated, and 4 were downregulated. Two genes involved in fatty acid uptake and 9 involved in lipid droplet biogenesis were upregulated, whereas 2 genes and 4 genes from the respective categories were downregulated, and 3 genes associated with β-oxidation were upregulated (Fig. 1e and Table S2). We further confirmed the upregulation of two lipases, *Pnpla2* (1.4-fold) and *Mgll* (2.5-fold); two fatty acid uptake genes, *Cd36* (1.6-fold) and *Fabp5* (1.9-fold); and one gene from the β-oxidation pathway, *Cpt1* (1.5-fold) and *Dgat2* (2.1-fold), involved in lipid droplet biogenesis in differentiated osteoblasts by qRT‒PCR (Fig. 1f). Interestingly, although *Cpt1*, *Pnpla2* and *Dgat2* upregulation did not reach statistical significance in RNA sequencing analysis, it did in qRT‒PCR validation. Upregulation of *Pnpla2*, the gene that encodes ATGL, the first enzyme in the cytosolic lipolysis pathway, was further confirmed by qRT‒PCR from an additional independent experiment (Fig. S2). The primer efficiencies of *Pnpla2* and housekeeping gene *Hprt1* (Table S3) along with their melt curve peaks were further validated (Fig. S2). *Hprt1* was used as a housekeeping gene, as its expression in the cells remained constant throughout differentiation (average C_t_ values 21.7 and 22.1 on Day 0 and Day 7, respectively, from 3 independent experiments).

### Inhibiting ATGL leads to blockade of lipolysis and accumulation of larger lipid droplets in mature osteoblasts

**Fig. 2:**
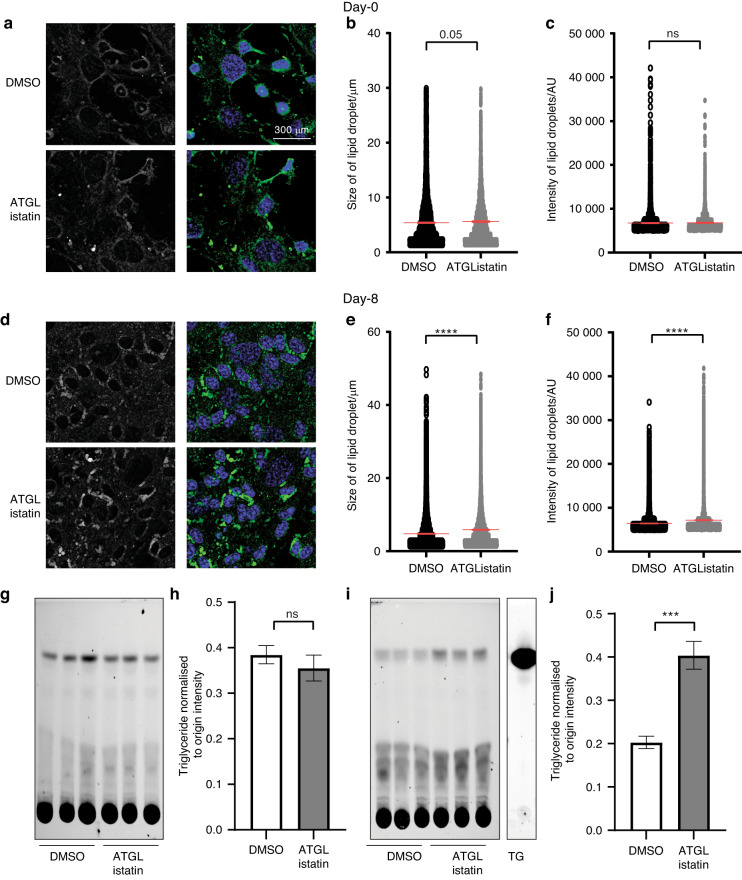
Effect of blocking ATGL on lipid metabolism in osteoblasts. Representative confocal images of **a** undifferentiated stromal cells on the 0^th^ day and **d** differentiated osteoblasts on the 8^th^ day of ex vivo differentiation from mice. Cellular lipid droplets were stained with BODIPY 493/503 (green in the merged panel), and the cells were mounted with DAPI (blue in the merged panel) in the presence or absence of ATGListatin. Panel 1 shows monochrome images of lipid droplets, whereas Panel 2 shows a merged image. Quantification of **b** size and **c** intensity in stromal cells and **e** size and **f** intensity in osteoblasts of BODIPY 493/503-stained lipid droplets in DMSO-treated control cells (open circle) and ATGListatin-treated cells (gray closed circle), where each dot represents the size or intensity of one lipid droplet. The data are representative of 3 independent experiments (*n* = 3) and are the mean ± standard error of the mean. The size or intensity of each lipid droplet was measured from independent images captured in 6 different fields of view of the coverslip (*n* = 6) for each experiment. *t* tests were performed assuming a normal distribution since the data points were more than 40 to determine significance between two groups where *P < 0.05, **P < 0.01, ***P < 0.001, ****P < 0.000 1. Thin-layer chromatogram of lipids harvested from BODIPY 558/568 C_12_ (red fluorescent fatty acid)-labeled **g** stromal and **i** ex vivo-differentiated mature osteoblast cells in mice, along with the chromatogram of fluorescently labeled triglyceride run as the standard. Densitometric quantification of labeled triglyceride normalized to origin in **h** stromal cells and in **j** mature osteoblasts in the presence (gray bar) or absence (open bar) of ATGListatin. The data are representative of 3 independent experiments (*n* = 3) and are presented as the mean ± standard deviation of 3 wells (*n* = 3). *t* tests or nonparametric Mann‒Whitney tests were performed accordingly after testing normal distribution using the Shapiro‒Wilk normality test to determine significance between two groups, where *P < 0.05, **P < 0.01, ***P < 0.001, ****P < 0.000 1

Given the upregulation of cytosolic lipases *Pnpla2* and *Mgll*, along with the presence of enhanced lipid droplets in mature osteoblasts compared to undifferentiated stromal cells, we asked whether cytosolic lipolysis regulates lipid metabolism during osteoblastogenesis. The first enzyme involved in cytosolic lipolysis, ATGL, can be potently and specifically inhibited with ATGListatin, a small molecule inhibitor.^18,19^ Therefore, we exploited the potential of ATGListatin to determine how cytoplasmic lipolysis impacts osteoblast differentiation and function. ATGListatin was used at a concentration of 50 μmol·L^−1^, as this concentration did not impact cell viability (Fig. S3). Confocal imaging showed no change in lipid droplet puncta in undifferentiated stromal cells treated with ATGListatin (Fig. 2a). Further quantification confirmed no change in lipid droplet size (Fig. 2b) or content measured in terms of the intensity of BODIPY 493/503 (Fig. 2c). However, compared to no ATGListatin treatment, treatment with ATGListatin led to accumulation of larger lipid droplets in mature, differentiated osteoblasts (Fig. 2d), as measured by both increased size (5.86 ± 0.048 μm versus 4.75 ± 0.039 μm) (Fig. 2e) and increased intensity of BODIPY493/503 staining (7 164 ± 33.53 AU versus 6 418 AU ± 17.62 AU) (Fig. 2f). Furthermore, a pulse chase assay was performed using the red fluorescence-labeled fatty acid BODIPY 558/568 C_12_ (Red C_12_) to measure lipolysis, which showed no changes in stromal cells (Fig. 2g, h) in the presence of ATGListatin but accumulation of Red C_12_ in lipid esters only in mature osteoblasts (Fig. 2i, j) (0.403 9 ± 0.03 AU versus 0.203 1 ± 0.014 AU). These data further confirm that while inhibiting lipolysis in undifferentiated stromal cells has minimal impact on lipid accumulation, perturbation of this pathway in mature osteoblasts distinctly alters the intracellular lipid profile.

### Inhibition of ATGL-mediated lipolysis impairs osteoblast maturation and activity

**Fig. 3:**
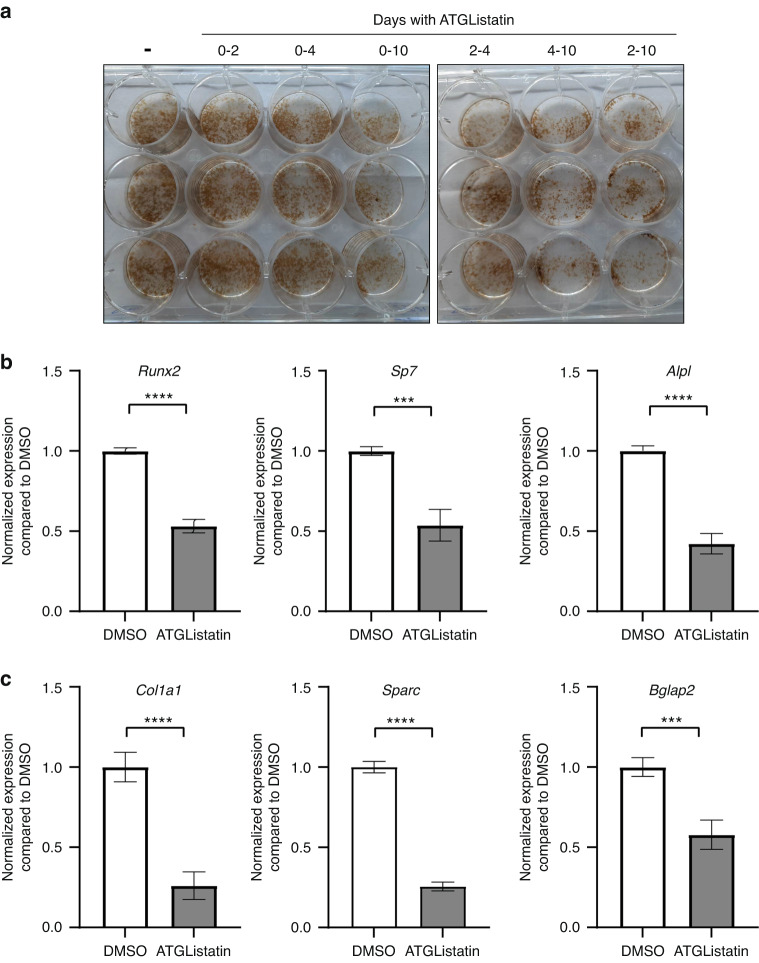
Effect of blocking ATGL on osteoblast maturation and functioning. **a** Representative image of von Kossa staining of ex vivo-differentiated osteoblasts on the 10^th^ day in the presence or absence of ATGListatin for different time frames. The image is representative of 3 independent experiments (*n* = 3). Quantitative real-time PCR-based expression data of **b** early and **c** late differentiation marker genes in the presence of DMSO (open bar) or ATGListatin (gray bar) in osteoblasts differentiated on the 10^th^ day in which ATGListatin was present throughout differentiation in the ATGListatin-treated group. The fold change compared to DMSO was calculated by normalizing the housekeeping gene normalized expression of individual genes in the treated group to the mean of housekeeping gene normalized expression of those genes in the DMSO control group. The data are the mean ± standard error of the mean pooled from 3 independent experiments (*n* = 3), each having 3 technical replicates (*n* = 9). *t* tests or nonparametric Mann‒Whitney tests were performed accordingly after testing normal distribution using the Shapiro‒Wilk normality test to determine significance between two groups, where *P < 0.05, **P < 0.01, ***P < 0.001, ****P < 0.000 1

To investigate how altering cytosolic lipolysis affects osteoblast function, osteoblasts were incubated with or without ATGListatin during various stages of osteoblastogenesis, and mineralization was stained using von Kossa staining (Fig. 3a). More specifically, BMSCs/osteoblasts were incubated in the presence of ATGListatin for different durations. Inhibition of lipolysis using ATGListatin during the entire duration of differentiation (0-10 days) resulted in a dramatic reduction in mineralization compared to osteoblast differentiation in the absence of ATGListatin (Fig. 3a). Interestingly, blocking lipolysis at later stages of differentiation (2-10 and 4-10 days) reduced osteoblast mineralization compared to controls, whereas ATGListatin treatment earlier in differentiation (0-2 or 0-4 days) had minimal impact on osteoblast mineralization (Fig. 3a). We further confirmed the disruption of osteoblastogenesis and activity during the continuous presence of ATGListatin throughout differentiation, as evidenced by downregulation of the differentiation markers *Runx2*, *Sp7* (1.9-fold), and *Alpl* (2.4-fold) (Fig. 3b) as well as late differentiation markers, including secretory proteins collagen encoded by *Col1a1* (3.8-fold), osteonectin encoded by *Sparc* (3.9-fold) and osteocalcin encoded by *Bglap2* (1.75-fold), in cells that were kept in osteogenic medium for 10 days (Fig. 3c). *Hprt1* was used as a housekeeping gene for normalization because its expression was not affected by ATGListatin treatment in osteoblasts (average C_t_ values of 22.1 and 21.9 in DMSO- and ATGListatin-treated cells, respectively, from 3 independent experiments).

### Cytosolic lipolysis-derived fatty acids serve as fuel to generate mitochondrial ATP for mature osteoblasts

**Fig. 4:**
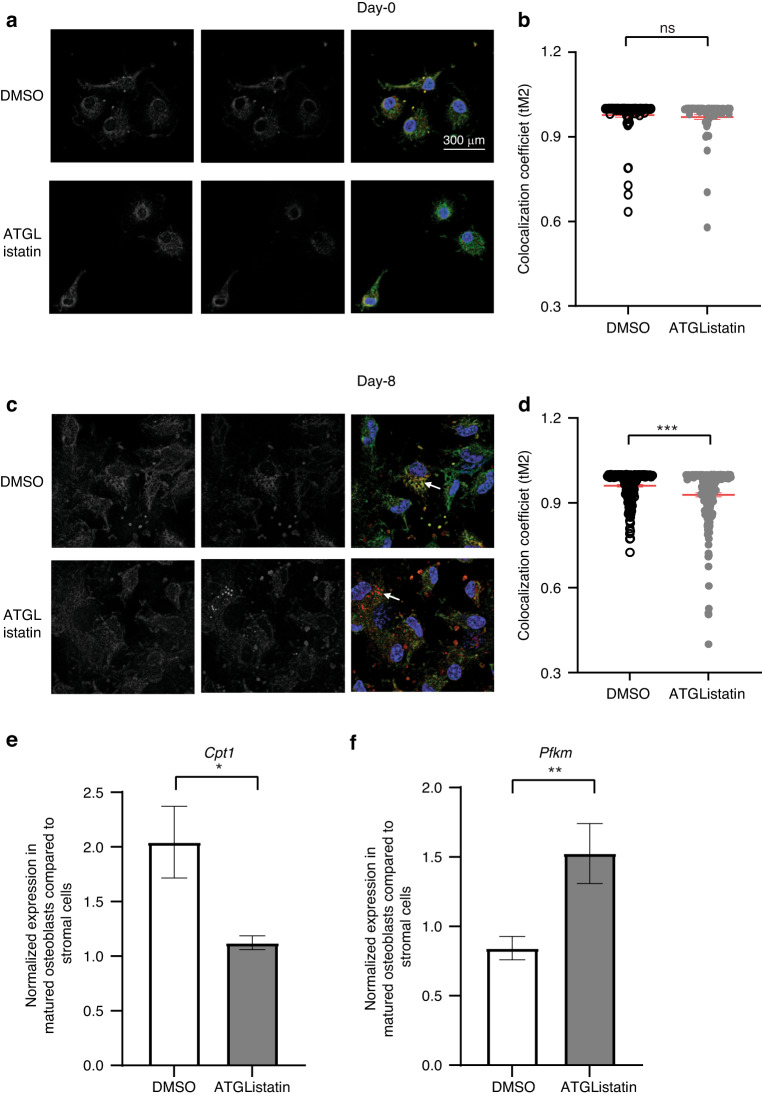
Effect of blocking ATGL on mitochondrial fatty acid availability. Representative confocal image of BODIPY 558/568 C_12_-labeled cells in which mitochondria were stained with mitodsRed and the cells were mounted with DAPI in the presence or absence of ATGListatin in **a** undifferentiated stromal cells on the 0^th^ day and **c** differentiated osteoblasts on the 8^th^ day of ex vivo differentiation. Panels 1 and 2 show monochrome images of mitochondria and BODIPY 558/568 C_12,_ respectively, whereas Panel 3 is the merged image where mitochondria are pseudocolored green, BODIPY 558/568 C_12_ is red and DAPI blue. Quantification of colocalization of BODIPY 558/568 with MitodRed in terms of threshold-adjusted Mander’s coefficient (tM2) in the absence (open circle) or presence (gray circle) of ATGListatin in **b** undifferentiated stromal cells on the 0^th^ day and **d** differentiated osteoblasts. The data are the mean ± standard error of the mean (SEM) pooled from 3 independent experiments (*n* = 3). Each dot represents colocalization measured per cell counted from independent images captured in 6 different fields of view in each experiment (*n* = 18). *t* tests were performed assuming a normal distribution since there were more than 40 data points used to determine significance between two groups, where *P < 0.05, **P < 0.01, ***P < 0.001, and ****P < 0.000 1. Analysis was performed on a single stack using Fiji ImageJ. **e** Fold change in quantitative real-time PCR-derived normalized expression of the β-oxidation gene *Cpt1* and **f** glycolytic gene *Pfkm* on the 10^th^ day of differentiation compared to the 0^th^ day of differentiation in stromal cells in the absence (open bar) or presence (gray bar) of ATGListatin. The data are the mean ± standard error of the mean pooled from 3 independent experiments (*n* = 3), each having 3 technical replicates (*n* = 9). *t* tests or nonparametric Mann‒Whitney tests were performed accordingly after testing normal distribution using the Shapiro‒Wilk normality test to determine significance between two groups, where *P < 0.05, **P < 0.01, ***P < 0.001, ****P < 0.000 1

Confocal imaging was performed following a pulse chase experiment in which Red C_12_ was pulsed in and then chased to determine colocalization with the mitochondria using Mito DsRed in the absence or presence of ATGListatin in both stromal cells and mature osteoblasts. There was no observed difference in colocalization of the red C_12_ fatty acids with the mitochondria (pseudocolored green) between the two groups in stromal cells (Fig. 4a). This was further quantified by the threshold-adjusted Manders’ colocalization coefficient (tM2) (Fig. 4b). Interestingly, red C_12_ showed colocalization with mitochondria under control conditions in mature osteoblasts (shown with arrowhead Fig. 4c). However, impairing lipolysis in mature osteoblasts with ATGListatin showed that Red C_12_ was present in puncta and did not colocalize with mitochondria (shown with arrowhead Fig. 4c). Further quantification revealed significantly decreased colocalization measured in terms of the threshold-adjusted Manders’ coefficient in mature osteoblasts after 7 days in the presence of ATGListatin (0.960 6 ± 0.004 in DMSO compared to 0.928 2 ± 0.007 in ATGListatin-treated cells) (Fig. 4d). Additionally, we observed twofold higher expression of *Cpt1* during normal osteoblastogenesis (Fig. 4e). Interestingly, however, in the presence of ATGListatin, the expression remained unaltered throughout osteoblast differentiation (Fig. 4e). Whereas, the expression of *Pfkm*, the rate-limiting enzyme of glycolysis, increased during osteoblast differentiation, with mature osteoblasts showing 1.5-fold higher expression than stromal cells in the presence of the lipolysis inhibitor, but in the DMSO-treated group, its expression remained the same (Fig. 4f).

**Fig. 5:**
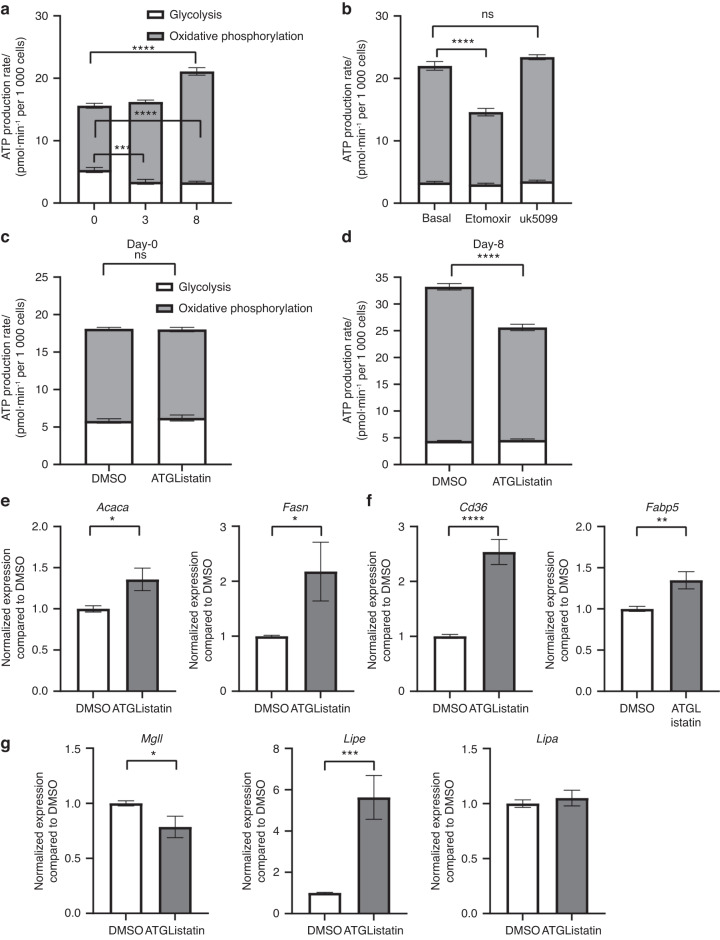
Effect of blocking ATGL on osteoblast energetics. ATP produced by glycolysis (white) versus oxidative phosphorylation (shaded) as measured by Seahorse ATP rate assay in the absence of any external nutrients **a** during ex vivo osteoblastogenesis at the indicated time points. **b** In differentiated matrix-secreting osteoblasts on the 8^th^ day of ex vivo differentiation in the absence or presence of etomoxir (Eto) and UK-5099 (UK). in the absence or presence of ATGListatin in **c** 0-day stromal cells and **d** 8-day-differentiated osteoblasts. The data are representative of 3 independent experiments (*n* = 3) and are the mean ± standard error of mean of data normalized to cell counts per well with data from a minimum of 11 wells per group (*n* = 11). Quantitative real-time PCR-based expression of **e** de novo fatty acid synthesis, **f** fatty acid uptake, and **g** lipase genes in the presence of DMSO (open bar) or ATGListatin (gray bar) in 10-day-differentiated osteoblasts in which ATGListatin was present throughout in the ATGListatin-treated group. The fold change compared to DMSO was calculated by normalizing the housekeeping gene normalized expression of individual genes in the treated group to the mean of housekeeping gene normalized expression of that gene in the DMSO control group. The data are the mean ± standard error of the mean pooled from 3 independent experiments (*n* = 3), each having 3 technical replicates (*n* = 9). *t* tests or nonparametric Mann‒Whitney tests were performed accordingly after testing normal distribution using the Shapiro‒Wilk normality test to determine significance between two groups, where *P < 0.05, **P < 0.01, ***P < 0.001, ****P < 0.000 1

We next sought to determine whether fatty acids released upon lipolysis and shuttled to the mitochondria were in fact being oxidized for ATP generation in osteoblasts. Interestingly, in the absence of exogenous substrates, undifferentiated stromal cells (0 day) relied on glycolysis to produce ATP (5.3 ± 0.4 pmol·min^−1^) from endogenous substrates compared to the cells committed toward osteoblasts obtained from the 3^rd^ day of ex vivo osteoblastogenesis (3.4 ± 0.4 pmol·min^−1^) or mature osteoblasts from the 8^th^ day of differentiation (3.3 ± 0.2 pmol·min^−1^) (Fig. 5a). Mature osteoblasts utilized oxidative phosphorylation more to generate ATP (17.8 ± 0.6 pmol·min^−1^) than undifferentiated stromal cells (10.3 ± 0.4 pmol·min^−1^) (Fig. 5a). To determine the intracellular nutrient source or the substrate fueling oxidative phosphorylation in mature osteoblasts, ATP generation was calculated in the absence or presence of UK5099, an inhibitor of mitochondrial pyruvate carrier, or etomoxir, a Cpt1 inhibitor. We observed that blocking glucose entry into the mitochondria using UK5099 was unable to inhibit oxidative phosphorylation-mediated ATP generation in mature osteoblasts (Fig. 5b). However, inhibiting long-chain fatty acid transport into the mitochondria using etomoxir decreased oxidative phosphorylation from 18.70 ± 0.70 pmol·min^−1^ to 11.60 ± 0.60 pmol·min^−1^ (Fig. 5b). As expected, none of the inhibitors affected glycolytic ATP production. Interestingly, blocking β-oxidation by etomoxir also led to the accumulation of cytosolic lipid droplets in ex vivo-differentiated osteoblasts, as observed by the increased lipid droplet size and intensity of BODIPY staining (Fig. S4a). Blocking lipolysis with ATGListatin did not affect the oxidation of stromal cells on the 0^th^ day (Fig. 5c) but was able to inhibit ATP generation via oxidative phosphorylation in mature osteoblasts (8^th^ day of differentiation), decreasing ATP generation from 28.8 ± 0.6 pmol·min^−1^ to 21.0 ± 0.6 pmol·min^−1^ (Fig. 5d). These data indicate that under nutrient-depleted conditions, fatty acids released by cytosolic lipolysis fuel ATP production by oxidative phosphorylation in mature osteoblasts, whereas stromal cells, which are more dependent on glycolysis, are unresponsive to this process. Blocking mitochondrial fatty acid transfer with etomoxir was able to further decrease oxidative phosphorylation-mediated ATP production compared to ATGListatin-mediated inhibition. However, cotreatment with both of these drugs did not decrease ATP production further compared to etomoxir alone (Fig. S4b). We further noted increased expression of the de novo fatty acid synthesis genes *Acaca* (1.4-fold) and *Fasn* (2.1-fold) (Fig. 5e), as well as the fatty acid uptake genes *Cd36* (2.5-fold) and *Fabp5* (1.3-fold) (Fig. 5f), in the presence of ATGListatin, suggesting enhanced availability of other potential fatty acid sources. The elevated expression of *Dgat1* and *Dgat2*, genes involved in lipid droplet biogenesis in the presence of ATGListatin (Fig. S5), suggests that a proportion of these fatty acids from other sources are packaged as lipid droplets. This phenomenon, along with blocked lipolysis, could explain the increased lipid droplet size in the presence of ATGListatin. Although the availability of exogenous fatty acids was not able to increase oxidative phosphorylation-mediated ATP generation in stromal cells or differentiated osteoblasts, it was able to compensate for the reduced ATP generation in the presence of ATGListatin (Fig. S6a, b). This indicated that the increased expression of fatty acid uptake genes in the presence of ATGListatin was a response to the inhibition of lipolysis and can help cells recover in terms of energy production in the presence of exogenous fatty acids. This further verifies the importance of fatty acid oxidation in differentiated osteoblasts. However, the presence of exogenous glucose during nutrient-replete conditions was not able to compensate for the loss of lipolysis-mediated ATP production. Blocking lipolysis with ATGListatin was able to decrease oxidative phosphorylation-mediated ATP even in the presence of exogenous glucose at both physiological concentrations (5 mmol·L^−1^) and at high concentrations typically used for in vitro culture systems (10 mmol·L^−1^) (Fig. S6c). Interestingly, although stromal cells can be treated with exogenous fatty acids (oleic acid) to ‘lipid-load’, this phenomenon does not result in increased ATP production under normal conditions (Fig. S6d, e). Moreover, the expression of *Mgll* encoding MGL was moderately reduced (1.3-fold), whereas that of *Lipe* encoding HSL was upregulated (5.6-fold) (Fig. 5g), in the presence of ATGListatin. The expression of *Lipa*, encoding lysosomal lipase (LAL), remained unchanged (Fig. 5g). This upregulation of *Lipe* could be a compensatory mechanism comparable to the hydrolysis of fatty acids from TAGs during the inhibition of ATGL.

**Fig. 6:**
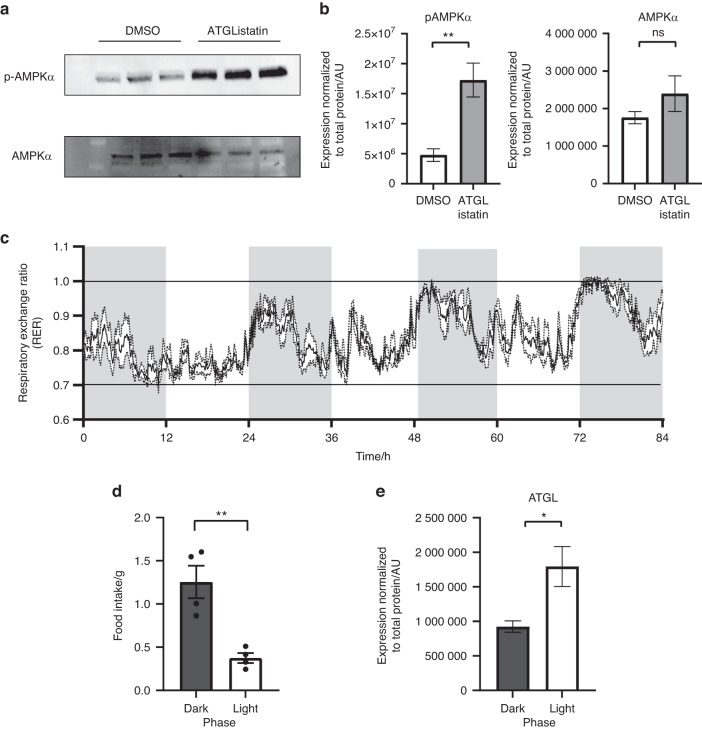
Physiological relevance of ATGL-mediated metabolism to osteoblast energetics. **a** Immunoblotting of ex vivo-differentiated osteoblasts with anti-pAMPKα (Thr172) and AMPKα in the presence or absence of ATGListatin. **b** Quantification of pAMPKα and AMPKα expression normalized to total protein in respective lanes measured by no-stain protein labeling in the absence (open bar) or presence of ATGListatin (gray bar). The data are the means ± standard deviations from three wells. **c** Respiratory exchange ratio of C57BL6/N mice over an 84-h (3.5-day) time period. Shaded vertical bars indicate a 12-h dark phase/cycle, and white vertical bars indicate a 12-h light phase/cycle. **d** Average daily food intake by these mice during the dark (dark gray bar) and light phases (open bar). The data are the means ± standard errors of the means, where *n* = 4 animals. **e** Quantification of ATGL expression in the femur cortex during the dark (dark gray bar) and light phases (open bar) normalized to total protein in respective lanes as measured by no-stain protein labeling. The data are the means ± standard errors of the means from *n* = 3 and *n* = 2 animals in dark and light cycles, respectively

Reduced production of ATP due to ATGListatin-mediated blockade of lipolysis was further confirmed by increased expression of pAMPKα (Thr172) (1.7×10^7^ ± 2.8×10^6^ AU in ATGListatin-treated cells compared to 4.7×10^6^  ± 6.0×10^5^ AU in DMSO-treated cells) without any effect on the expression of pan AMPKα (Fig. 6a, b). Given these data, it stands to reason that during instances of depleted exogenous nutrients, lipolysis within the osteoblast could be a mechanism by which bone formation is maintained. Therefore, to provide physiological context, we confirmed that mice rely on fatty acid oxidation during their light/inactive phase (RER is ~0.7) (Fig. 6c) when they do not actively eat (Fig. 6d). However, during their dark/active phase, while they are ingesting food, whole-body metabolism switches to utilizing more carbohydrate substrates (RER > 0.7) (Fig. 6c). We further showed increased expression of ATGL protein in the bone cortex of mice during their light cycle (1.8×10^6^ ± 2.9×10^5^ AU) compared to that during their dark cycle (9.2×10^5^ ± 8.2×10^4^ AU) (Fig. 6e). These data support the tenet that fatty acids are utilized by mice during unfed conditions and that lipid droplets serve as this fatty acid source in osteoblast cells.

### Knocking down *Pnpla2* in osteoblast progenitor cells in mice leads to loss of bone mass without affecting central metabolism

**Fig. 7:**
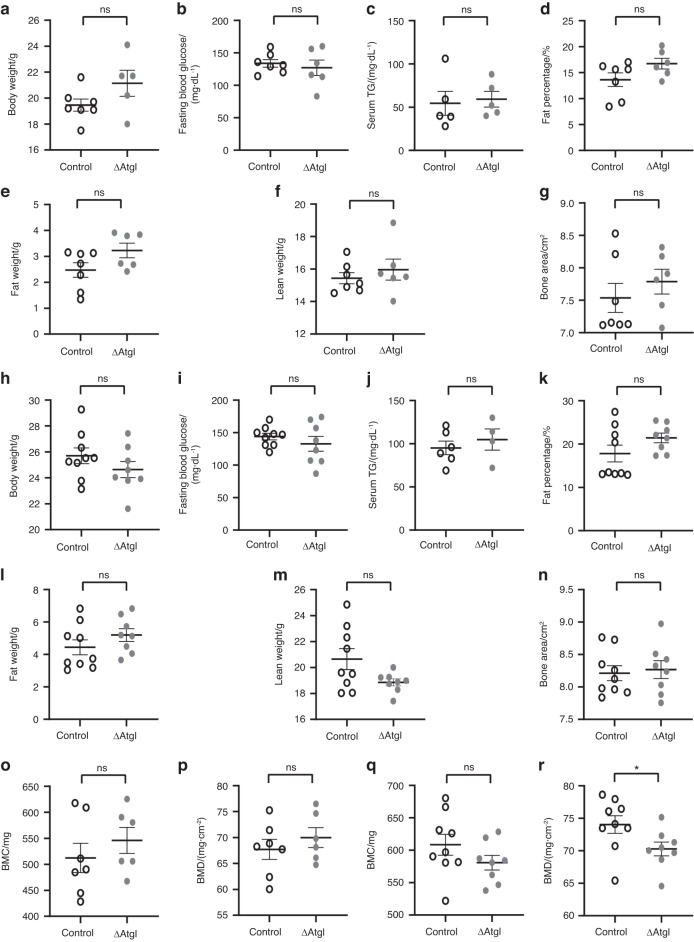
Effect of conditionally knocking out the ATGL coding gene in osteoblast precursor cells (ΔATGL) on central metabolism. **a** Body weight, **b** fasting blood glucose, and **c** serum triglyceride (TG) levels of 12-week-old control (open circle) and ΔATGL (closed gray circle) female mice. **d** Fat percentages, **e** fat weights, **f** lean weights, and **g** bone areas of 12-week control (open circle) and ΔATGL female mice (closed gray circle) as measured by DXA scanning. **h** Body weight, **i** fasting blood glucose, and **j** serum triglyceride (TG) levels of 12-week-old control (open circle) and ΔATGL (closed gray circle) male mice. **k** Fat percentages, **l** fat weights, **m** lean weights, and **n** bone areas of 12-week control (open circle) and ΔATGL male mice (closed gray circle) as measured by DXA scanning. **o** Bone mineral content (BMC; mg) and **p** bone mineral density (BMD; mg/cm^2^) as measured by DXA scanning in 12-week-old female and male **q**, **r** control (open circle) and ΔATGL (closed gray circle) mice. Each dot represents data from individual animals, where *n* = 7 in control and 5/6 in knockout females and *n* = 9 in control and 8 in knockout males, except serum TG, where *n* = 5 for both control and knockout females and *n* = 6 and 4 for control and knockout males. The data are the mean ± standard error of the mean. *t* tests or nonparametric Mann‒Whitney tests were performed accordingly after testing normal distribution using the Shapiro‒Wilk normality test to determine significance between two groups, where *P < 0.05, **P < 0.01, ***P < 0.001, ****P < 0.000 1

To confirm the role of lipolysis in supporting osteoblast activity in vivo, we generated mice in which the *Pnpla2* gene (ATGL) was conditionally disrupted in osteoblast progenitor cells of the long bones. Pnpla2^loxp/loxp^ mice in which exons 2 through 7 were flanked by loxP sites were crossed with Prx1-Cre mice to generate osteoblast-specific mutants (Prx1-Cre^TG/+^;Pnpla2^lox/lox^, (hereafter referred to as ΔATGL) and control/wild-type littermates (Pnpla2^loxp/loxp^). Both male and female ΔATGL mice were born at the expected Mendelian frequency. The osteoblast progenitor-specific knockout mice exhibited 1.7-fold reductions in *Pnpla2* mRNA levels in the bone cortex (devoid of marrow) with normal expression in other metabolic tissues (Fig. S7a, b). Both male and female mice exhibited knockout of ATGL in terms of protein expression in the bone cortex (Fig. S7c, d). No changes were reported in body weight, fasting blood glucose, serum triglycerides, body fat percentage or fat and lean whole-body mass in either female (Fig. 7a-f) or male (Fig. 7h-m) knockout mice. Additionally, no changes were detected in the bone area with knockout in mice of either sex (Fig. 7g, n). Although there were no changes in either bone mineral content (BMC) (Fig. 7o) or bone mineral density (BMD) in female mice (Fig. 7p), BMC in the ΔATGL male mice showed a decreasing trend (Fig. 7q) (decreased from 608.5 ± 16.16 mg to 580.8 ± 11.32 mg), and the BMD decreased from 74.04 ± 1.357 mg·cm^−2^ to 70.28 ± 1.076 mg·cm^−2^ (Fig. 7r) in the ΔATGL male mice compared to controls. No change was detected in longitudinal bone growth in male mice, as indicated by tibia length (17.61 ± 0.16 mm in control mice and 17.47 ± 0.13 mm in ΔATGL mice). We further confirmed no alteration in other serum lipid profiles, including cholesterol or fatty acids (Fig. S8a) or adipocyte number or volume in epididymal white adipose tissue (eWAT) (Fig. S8b), between the genotypes. Although we did observe a decrease in adipocyte number and an increase in volume in the subcutaneous WAT (subWAT) (Fig. S8c),^20,21^ the lack of alterations in other parameters (i.e., body weight, fat weight or fasting blood glucose) supported the idea that genetic manipulations do not affect central fat metabolism/systemic metabolism.

### Loss of ATGL function in osteoblast progenitor cells results in a low-bone-mass phenotype driven by reduced bone formation

**Fig. 8:**
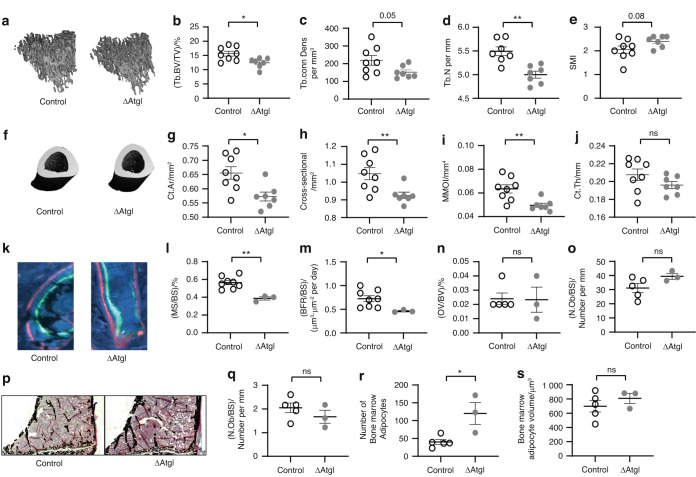
Bone health in ΔATGL mice. **a**–**e** Micro-computed tomography (µCT) analysis of the trabecular bone of the tibia in 12-week-old control (open circle) and ΔATGL mice (closed gray circle). **a** Representative 3D micro-CT image of trabecular bone. **b** Percentage bone volume over total volume [(Tb.BV/TV)/%]. **c** Connection density (Tb.conn Dens per mm^3^). **d** Trabecular number (Tb.N per mm). **e** Trabecular structure model index (Tb.SMI; 0= plates, 3= rods). **f**–**j** Micro-computed tomography (µCT) analysis of cortical bone. **f** Representative 3D micro-CT image of trabecular bone. **g** Cortical bone area (Ct.Ar/mm^2^). **h** Total cross-sectional area (Cross-sectional/mm^2^). **i** Minimum moment of inertia (MMOI/mm^4^). **j** Thickness (Ct.Th/mm). *n* = 8 in the control group and 7 in the knockout group. **l**–**o** Histomorphometric analysis of the tibia in 12-week-old control (open circle) and ΔATGL mice (closed gray circle). k-m) Histodynamic analysis. **k** Representative image of a bone section double-labeled with Alizarin (red) and Calicin (green). **l** Percentage mineralization surface over bone surface [(MS/BS)/%]. **m** Bone formation rate over bone surface. *n* = 8 in the control group and 3 in the knockout group [(BFR/BS)/μm^3^·μm^−2^ per day]. (**n**-**s**) Histostatic analysis. **n** Percentage osteoid volume over bone volume [(OV/BV)/%]. **o** Number of osteoblasts over the bone surface [(N.Ob/BS)/Number per mm]. **p** Representative image of a trichrome-stained bone section. **q** Number of osteoclasts over the bone surface [(N.Ob/BS)/Number per mm]. **r** Number of bone marrow adipocytes. **s** Volume of bone marrow adipocytes. *n* = 5 in the control group and 3 in the knockout group. All the data are presented as the mean ± standard error of the mean. *t* tests or nonparametric Mann‒Whitney tests were performed accordingly after testing normal distribution using the Shapiro‒Wilk normality test to determine significance between two groups, where *P < 0.05, **P < 0.01, ***P < 0.001, ****P < 0.000 1

Reduced trabecular bone was evident in the tibial metaphysis of the ΔATGL male mice (Fig. 8a), as indicated by significantly lower BV/TV [(12.45 ± 0.66)% compared to (15.64 ± 0.85)% of the control mice] (Fig. 8b), connectivity density [Conn.Dens; (152.1 ± 13.07) per mm^3^ versus (218.6 ± 27.12) per mm^3^] (Fig. 8c), and trabecular number [Tb.N; (5.00 ± 0.072) per mm versus (5.49 ± 0.092) per mm] (Fig. 8d). A trend toward lower trabecular thickness and tissue mineral density (TMD) and higher trabecular separation was noted in ΔATGL male mice, but these parameters were not statistically significant (Fig. S9a). The structure model index (SMI) trended toward 3.0 in the male ΔATGL mice (Tb.SMI; 2.4 ± 0.091 compared to 2.05 ± 0.155 in control mice), indicating ‘weaker bone’ and more rod-like bone (Fig. 8e). Micro-CT analyses of the tibia diaphysis revealed that ΔATGL mice had reduced cortical bone parameters as well (Fig. 8f), as indicated by a reduced cortical area (Ct.Ar; 0.572 ± 0.015 mm^2^), total cross-sectional area (Cross-sectional; 0.92 ± 0.017 mm^2^), and minimum moment of inertia (MMOI; 0.049 5 ± 0.001 7 mm^4^) compared to those in control mice (Ct.Ar; 0.655 ± 0.021 mm^2^, cross-sectional; 1.047 ± 0.034 mm^2^ and MMOI; 0.063 4 ± 0.003 4 mm^4^) (Fig. 8g–i). Although the cortical thickness was slightly lower in the male ΔATGL mice (Ct.Th; 0.196 0 ± 0.003 8 mm versus 0.207 6 ± 0.006 mm) (Fig. 8j), it was not statistically significant. No differences were noted in marrow volume, cortical tissue mineral density or porosity (Fig. S9b). As expected, given the PrrX1 promoter-targeted deletion, we did not find any changes in vertebral BV/TV between wild-type and ΔATGL mice (Fig. S9c). Tibias from female ΔATGL and wild-type mice were also examined for alterations in bone microarchitecture, but no differences were detected in trabecular cortical bone (Fig. S10a, b). Therefore, additional analyses were performed exclusively for male samples.

Bone histomorphometry analysis demonstrated reduced osteoblast activity (Fig. 8k), as indicated by lower mineralization surface per bone surface [MS/BS; (0.386 7 ± 0.018)%] and reduced bone formation rates (BFR/BS; 0.46 ± 0.015 μm^3^·μm^−2^ per day) in ΔATGL mice compared to control mice [MS/BS; (0.563 8 ± 0.023 6)%, BFR/BS; 0.723 846 ± 0.063 μm^3^·μm^−2^ per day] (Fig. 8l, m). Trichrome staining (Fig. 8p) showed no significant changes in osteoid volume per bone volume [OV/BV; (0.023 3 ± 0.008)% in ΔATGL and (0.024 ± 0.04)% in control mice] (Fig. 8n), number of osteoblasts, or osteoclasts on the bone surface (N.Ob/BS; 39.42 ± 2.06 number per mm and 31.17 ± 3.22 number per mm or N.Oc/BS; 1.670 ± 0.275 number per mm and 2.050 ± 0.201 0 number per mm in ΔATGL and control mice, respectively) (Fig. 8o and q). Knockdown of ATGL using Prrx1Cre also resulted in an increase in bone marrow adipocyte (BMAd) number (120 ± 30.35 versus 40 ± 6.85) (Fig. 8r) without any changes in volume (Fig. 8s).

### Knocking out ATGL in osteoblast progenitor cells alters lipid metabolism in bone

**Fig. 9:**
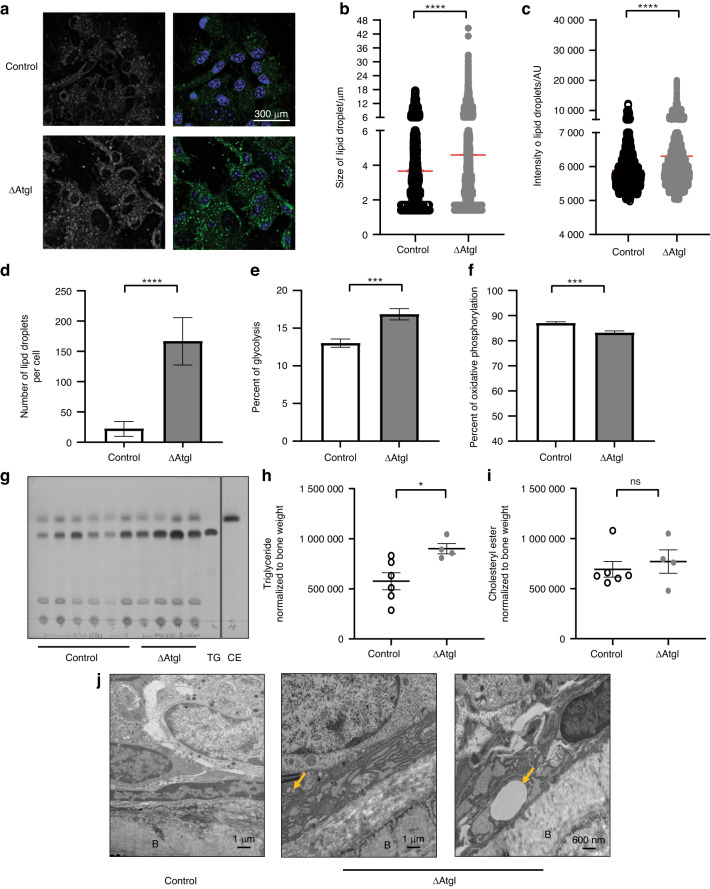
Lipid metabolism in bone and osteoblasts of ΔATGL mice. **a** Representative confocal image of ex vivo-differentiated osteoblasts on the 8^th^ day of differentiation from control and ΔATGL mice in which cellular lipid droplets were stained with BODIPY 493/503 (green in merged panel). Quantification of **b** size and **c** intensity of BODIPY 493/503-stained lipid droplets in differentiated osteoblasts from control (open circle) or ΔATGL mice (gray closed circle), where each dot represents the size or intensity of one lipid droplet. **d** Quantification of the number of lipid droplets per cell in differentiated osteoblasts from control (open) and ΔATGL mice (gray). **e**, **f** Seahorse ATP rate assay. Percentage of **e** glycolytic and **f** oxidative phosphorylation measured by Seahorse ATP rate assay in absence of any external nutrients in ex vivo*-*differentiated osteoblasts on the 8^th^ day of differentiation from 12-week-old control (open) and ΔATGL mice (gray) mice. **g** Thin-layer chromatogram of lipids harvested from flushed tibias (tibia without any bone marrow) from 12-week-old control and ΔATGL mice. Densitometric quantification of the lipid species from control (open circle) and ΔATGL mice (gray closed circle) from the chromatogram **h** triglyceride, **i** cholesteryl ester normalized to bone weight. **j** Transmission electron microscopy image of thinly sectioned tibia showing the ER (red arrow) and lipid droplets (yellow arrow) in the cytosol of osteoblasts positioned next to the bone surface (B) in control and ΔATGL mice. The control and first image from ΔATGL mice was taken at 2 700 × magnification, and the second image is from a higher-magnification zoomed-in section where the image was taken at 4 400X. All the data are represented from individual animals, where *n* = 6 control and *n* = 4 knockout mice, and presented as the mean ± standard error of the mean. *t* tests or nonparametric Mann‒Whitney tests were performed accordingly after testing normal distribution using the Shapiro‒Wilk normality test to determine significance between two groups where *P < 0.05, **P < 0.01, ***P < 0.001, ****P < 0.000 1

As observed during ATGListatin treatment, confocal imaging showed accumulation of larger lipid droplets in mature osteoblasts (Day 8 of differentiation) from ΔATGL mice (Fig. 9a) but not in stromal cells (Fig. S11a-d). This was quantified by both increased size (4.59 ± 0.03 μm) and intensity of BODIPY493/503 staining (6 304 ± 9.44 AU) compared to the lipid droplets from cells cultured from control mice (3.66  ± 0.05 μm size and 5 886 ± 13.81 AU intensity) (Fig. 9b & c, respectively). Furthermore, osteoblasts from ΔATGL mice also showed an increased number of lipid droplets per cell (166.5 ± 39.07 versus 22.18 ± 12.22) (Fig. 9d). These cells also showed altered metabolism in terms of increased glycolysis (16.85 ± 0.75)% and decreased oxidative phosphorylation (83.15 ± 0.75)% compared to osteoblasts from control mice [(13.01 ± 0.55)/% and (86.99 ± 0.55)/%, respectively] (Fig. 9e & f). Lipids harvested from the flushed tibia, i.e., from the bone cortex without bone marrow, showed altered lipid metabolism (Fig. 9g). Quantification of the lipid profile by TLC showed significant accumulation of triglycerides (1.6-fold) in bones from ΔATGL mice compared to wild-type controls (Fig. 9h). However, the level of cholesteryl esters, another neutral lipid store, remained unchanged (Fig. 9i). Transmission electron microscopy (TEM) imaging further confirmed the accumulation of lipid droplets in the cytosol of osteoblasts from ΔATGL mice, as shown with yellow arrowheads (Fig. 9j).

## Discussion

These data provide strong evidence that osteoblasts utilize endogenous fatty acids, released by lipolysis from intracellular lipid droplet stores, as substrates to support ATP production when exogenous substrates are limited. It is widely appreciated that healthy bone mass is dependent on nutrient availability.^22–25^ The requirement to synthesize and secrete extracellular matrix (ECM) proteins along with mineralization vesicles reinforces the need for osteoblasts to rely heavily on cellular energy status or bioenergetics. While previous studies have focused on glucose supporting osteoblast function,^12–14^ fatty acids are an energy-dense substrate source. In this regard, oxidation of a single molecule of palmitic acid (C_16_ fatty acid, most abundant fatty acid in humans) can produce 4 times more ATP than complete oxidation of one molecule of glucose. As such, in addition to osteoblasts utilizing glucose for aerobic glycolysis, fatty acids remain an attractive fuel source, specifically when energy demand is high. To this end, our laboratory has recently demonstrated that oxidative phosphorylation relies upon ATP generation in osteoblasts.^26^ Moreover, elegant studies have further demonstrated the importance of long-chain fatty acid oxidation^16,27^ in bone homeostasis. For example, targeted deletion of carnitine palmitoyltransferase 2 (CPT2), the obligate enzyme required to facilitate the transfer of fatty acids back to CoA from their respective carnitylated form to regenerate fatty acyl CoA for oxidation after their entry into the mitochondria, in osteoblasts (using osteocalcin; Ocn-Cre) leads to significant impairments in postnatal bone acquisition in female mice.^16^ Additionally, knockout of CD36, which functions in high-affinity tissue uptake of long-chain fatty acids (FAs), results in a low-bone-mass phenotype driven by impairments in osteoblastogenesis, although this mechanism remains to be confirmed in a tissue-specific manner.^28^ Therefore, osteoblast function relies in part on the utilization of fatty acid substrates for ATP generation and subsequent skeletal health. These findings have been seminal in elucidating how metabolic processes regulate bone formation; however, they have not elaborated the origins or sources of these fatty acids. In other tissues and cells, fatty acid substrates can be acquired (1) by de novo lipogenesis from glucose substrates; (2) from exogenous, dietary sources via chylomicron remnants; (3) by endogenous mobilization from adipocytes; and/or (4) by intracellular lipolysis of stored lipid droplets. While all these mechanisms could supply osteoblasts with fatty acid substrates for energy generation, the recent identification of lipid droplets within osteoblastic cells supports the potential for an important and readily available source.

Lipid droplets were first observed in normal bone as early as 1965, when Enlow and colleagues described them in osteoblasts within the Haversian canals.^29^ While lipid droplets in osteoblasts appear to be a relatively normal occurrence, steroid treatment, alcoholism, and aging increase their number.^30–32^ Our laboratory has previously confirmed the accumulation of lipid droplets during osteoblast differentiation; however, the function of these organelles remains unknown. The current study provides further confirmation that osteoblasts isolated from BMSCs or calvarium accumulate lipid droplets upon osteogenic differentiation. This does not appear to be an artifact, as undifferentiated cells maintained for a similar time in culture do not demonstrate these organelles. Additionally, cells with lipid droplets were RUNX2+ osteoblasts. Gene expression analysis revealed global alterations in the metabolism of all three major nutrients, glucose, lipids, and amino acids, at the transcript level throughout maturation as osteoblasts. The increased expression of genes involved in glycogen breakdown and glycolysis was consistent with previously published reports, demonstrating that glucose utilization is important for osteoblast differentiation.^12–14^ In terms of lipid metabolism, the upregulation of genes involved in both the anabolic arm of lipid metabolism (*Plin2*, *Plin4*, *Gk5*, *Lpin3*, *Dgat2*) and catabolic metabolism (*Cpt2*, *Pnpla2*, *Mgll*) indicated highly dynamic lipid metabolic flux in mature osteoblasts.

To further detail how lipid droplets support osteoblast function, we blocked one arm of lipid flux by impairing lipolysis. Cytosolic lipolysis occurs by sequential activity of three enzymes that catalyze the hydrolysis of lipids. In this manner, adipose triglyceride lipase (ATGL/*Pnpla2*), hormone sensitive lipase (HSL/*Lipe*) and monoacyl glycerol lipase (MGL/*Mgll*) directly act on lipid droplets by hydrolyzing triglycerides (TAGs), diglycerides (DAGs), and monoglycerides (MAGs), respectively, to release free fatty acids and glycerol.^33^ We found that inhibiting ATGL impacts the proper function of osteoblasts both ex vivo*/*in vitro and in vivo. Interestingly, inhibiting lipolysis in mature matrix-secreting osteoblasts, not in undifferentiated stromal cells, has a more pronounced impact on osteoblast function, as indicated by Von Kossa staining. As expected, we detected accumulation of larger lipid droplets in these mature osteoblasts treated with ATGListatin or harvested from ΔATGL mice, which has been reported in other cell types.^34^ In this capacity, blocking lipolysis decreased the availability of fatty acids shuttled to the mitochondria for oxidative phosphorylation and ATP generation. Therefore, these data provide direct evidence that lipid droplets serve as an ‘energy’ reservoir capable of providing endogenous fatty acid substrates for supporting bone formation by osteoblasts. Interestingly, blocking lipolysis with ATGListatin was not able to decrease oxidative phosphorylation-mediated ATP production to the same extent as etomoxir, indicating the existence of other sources of endogenous fatty acids in mature osteoblasts. Moreover, since β-oxidation is downstream of lipolysis as well as all other processes that may contribute to fatty acid availability, using both inhibitors does not have any further additive effect over etomoxir-mediated inhibition of ATP production.

However, since etomoxir was not able to completely inhibit oxidative phosphorylation-mediated ATP production, there are likely other endogenous sources available that can be used by cells as biofuel. For example, several amino acids can enter the TCA cycle directly by transamination and deamination reactions.^35,36^ Additionally, ketone body catabolism generates acetyl CoA, which can also be used to generate ATP via oxidative phosphorylation.^35,37^ Finally, short-chain fatty acids do not require CPT1 to enter the mitochondria and therefore can be readily oxidized.^38–40^ Consistent with these data, the elevated expression of the de novo fatty acid synthesis genes *Acaca* and *Fasn* and the fatty acid uptake genes *CD36* and *Fabp5* in the presence of ATGListatin suggested enhanced availability of other potential fatty acid sources. However, the elevated expression of triglyceride synthesis genes such as *Dgat1* and *Dgat2* indicated that a major portion of this fatty acid coming from other sources may have also been packaged in lipid droplets after conversion into triglycerides. This may have contributed to the increased size of lipid droplets in the presence of the lipolysis inhibitor. Furthermore, the inability of exogenously added fatty acids to increase oxidative phosphorylation-mediated ATP generation under normal conditions confirmed that both BMSCs and osteoblasts prefer to utilize endogenous fatty acids even in the presence of external fatty acids. In fact, BMSCs prefer to store fat instead of using it, as observed by the accumulation of cytosolic lipid droplets when they were cultured in the presence of oleic acid without any change in ATP production. Interestingly, although these cells accumulated as many lipid droplets as differentiated osteoblasts, they, unlike osteoblasts, were not able to utilize them to generate ATP, further supporting the importance of lipolysis in differentiated cells. Moreover, inhibition of lipolysis in osteoblasts resulted in a metabolic switch toward ATP generation via glycolysis, presumably to compensate for the reduction in ATP generated from oxidative phosphorylation. These data support the tenet that blocking lipolysis in osteoblasts impairs fatty acid oxidation and that while cells attempt to generate ATP via glycolysis, this switch is unable to fully compensate for ATP lost due to reduced mitochondrial respiration. AMPKα can act as a sensor to detect energy stress, where it is phosphorylated when ATP availability is reduced and AMP presence is increased to enable cells to adapt to low energy availability.^41–43^ Increased phosphorylation of AMPKα (Thr172) without any increase in the expression of total AMPKα further confirmed that inhibiting lipolysis with ATGListatin decreases cytosolic ATP availability in osteoblasts. Phosphorylation of AMPKα further reprograms cells to minimize energy consumption to reserve available energy for functions essential for survival.^42^ This may explain why blocking lipolysis highly affected energy-demanding processes such as mineralization and differentiation of osteoblasts, as observed by lower Von Kossa staining and lower expression of differentiation marker genes.

It should be noted that unlike many of the previously performed metabolic flux studies,^26,44^ the assays executed in the current studies were performed in the absence of external nutrients (i.e., glucose, pyruvate, glutamine, or fatty acids), as doing so made cells more inclined to utilize endogenously stored nutrient sources for ATP generation. However, we further confirmed that the addition of only external fatty acids and not glucose was able to recover the decreased ATP production by ATGListatin. The addition of glucose even at a higher-than-physiological concentration in the external environment was not able to rescue the decreased ATP generation caused by inhibition of lipolysis. This confirms the importance of lipolysis for ATP generation by osteoblasts even under fed conditions. Interestingly, ATGL-mediated lipolysis becomes physiologically relevant for energy production in bones in the absence of exogenous nutrients, as observed by its increased expression in the mouse femur cortex during the light phase/day, when food intake is significantly lower. These mice also utilize more fat during the day (or light phase) for ATP generation when they are not eating.^45^ In summary, these data indicate that mice depend on fatty acids from ATGL-mediated lipolysis for ATP generation when they are not actively eating and have less availability of exogenous nutrients.

These molecular data were supported by the generation of a novel mouse model in which lipolysis was impaired via targeted deletion of *Atgl* in early osteoblasts of the limb bud. This promoter was selected to knock out gene expression early during osteoblast differentiation and throughout lineage commitment, although it is also expressed in marrow adipocytes and subcutaneous adipocytes.^20,21^ Decreased cell count and increased cell volume of subcutaneous adipose tissue indicated that ATGL in this tissue was potentially targeted. However, since we did not find any change in body weight, fat mass, fasting blood glucose, or serum lipid profiles, we are confident that knockout did not affect systemic metabolism, indirectly influencing bone. This was further confirmed due to the lack of a bone phenotype in the axial skeleton, which was not targeted using Prrx1-Cre. The defective maturation and mineralization noted in ex vivo osteoblasts were recapitulated in ΔATGL mice that showed impairment in bone formation rates, resulting in a low-bone-mass phenotype in male mice. No changes were detected in osteoblast or osteoclast number, underscoring that the primary pathway impacted osteoblast function/activity. We did not observe changes in whole body weight, epididymal fat depot weight or blood serum fat marker levels in gene-deleted mice. Consistent with previous reports in which liver- and cardiac muscle-specific gene deletion of ATGL resulted in lipid accumulation in those tissues,^46,47^ we also observed lipid accumulation in bone. TAG is the major substrate for ATGL,^33,48^ and the major lipid species in bone were found to be primarily affected by the deletion, showing accumulation. We further confirmed that the increased lipid in bone was a result of the accumulation of lipid droplets in osteoblasts by visualizing their increased presence in the cytosol of these cells. Interestingly, contrary to our study, a previous study reported no change in trabecular bone mass in ΔATGL mice in which the knockout was driven by OCN-Cre.^16^ However, this study was conducted on 12-week-old female mice. Importantly, there appears to be sexual dimorphism in ΔATGL mice, which could explain this discrepancy. As such, ΔATGL female mice did not show any impairment in bone formation, although the protein is expressed equally in both sexes in BMSCs and ex vivo-differentiated osteoblasts, and both showed comparable levels of knockout in the bone cortex. While the molecular mechanism responsible for sexual dimorphism remains to be elucidated, female mice have been reported to be more resistant to metabolic perturbations,^49^ mainly due to the ability of estrogen to increase lipid utilization.^50^ The presence of a higher lipid content and the ability to utilize exogenous fatty acids by ex vivo-differentiated osteoblasts under normal conditions in the presence of estrogen may explain the differential lipid metabolism in female mouse bone and, hence, the redundancy of ATGL in maintaining bone homeostasis. Other differences between this and the previous study included a difference in the age of the mice and the use of different Cre promoters. In both studies, bone microarchitecture was determined at a single timepoint; therefore, it is entirely reasonable that we may have missed the window at which differences in female bone occurred. Finally, it is worth noting through these studies that HSL can also hydrolyze TAGs, albeit to a lesser extent than ATGL.^51^ Hence, it remains possible that blocking ATGL, either by genetic manipulation or chemical inhibition, can maintain some level of functional lipolysis. Therefore, inhibition of ‘complete’ lipolysis is expected to have an even greater impact on osteoblast function.

In conclusion, the current study demonstrates that endogenous fatty acids, stored as lipid droplets, are metabolized via mitochondrial oxidation for ATP generation, supporting the energic demands of bone formation by osteoblasts. Moreover, the physiological relevance of this process is particularly important when exogenous nutrients are limited. As such, cytoplasmic lipolysis serves as an important process to support skeletal homeostasis. These data highlight a molecular metabolic pathway during normal osteoblastogenesis. Metabolic disorders related to impaired lipid metabolism, including type 2 diabetes mellitus^52–56^ and dyslipidemia,^57^ as well as Gaucher diseases^58^ and Niemann-Pick disease,^59^ have long been associated with skeletal dysfunction, resulting in increased fracture incidence. Our data suggest that the pathogenesis of these disorders may include impaired lipid droplet oxidation. Based on the current study, we propose that impaired utilization of fatty acids reduces osteoblast bioenergetic capacity, thereby suppressing ATP-dependent mechanisms such as bone formation.

## Materials and methods

### Animal models

The generation of mice with *loxP* sites flanking exons 2 through 7 of *the PNPLA2* gene has been previously described,^60^ and these mice were obtained from The Jackson Laboratories (Strain#024278). The Pnpla2^lox/lox^ mice were bred with our Prx1-Cre (Prx1-Cre^TG/+^) mice to knock down ATGL in early mesenchymal cells within the limb bud (ΔATGL). PCR analysis of genomic DNA from the ear or toe was used to confirm genotypes (primer sequence in Table S3). All mice were generated on a C57BL6/N background. During maintenance, the mice used for the conditional knockout study were kept under a standard 12-h light/dark cycle and had *ad libitum* access to a standard chow diet and water until 4 weeks of age. At 5 weeks of age, mice were fed a purified diet (D12450J, Research Diets) and maintained on this diet for 8 weeks. BMSCs and calvarial osteoblasts were isolated from 8- to 10-week-old C57BL6/N mice for all in vitro studies performed with or without ATGListatin. A cohort of male C57BL/6 N mice was used for metabolic cage profiling. For studies comparing light/dark cycles, male mice were sacrificed during their ‘dark’ cycle in the presence of red light. The mice were maintained in a clean environment, and all procedures were conducted in strict adherence to the Institutional Animal Care and Use Committee (IACUC) requirements at Vanderbilt University Medical Center (VUMC).

### Indirect calorimetry

Mice were individually placed in home cages in a 12 h light/dark cycle, temperature/humidity-controlled dedicated room located in the Vanderbilt MMPC (RRID: SCIR_021939). Energy expenditure measures were obtained by indirect calorimetry (Promethion, Sable Systems, Las Vegas, NV). The calorimetry system consisted of home cages with bedding equipped with water bottles and food hoppers connected to load cells for food and water intake monitoring. All animals had *ad libitum* access to a purified diet and water. The air within the cages was sampled through microperforated stainless steel sampling tubes that ensure uniform cage air sampling. Promethion utilized a pull-mode negative pressure system with an excurrent flow rate set at 2 000 mL·min^−1^. Water vapor was continuously measured, and its dilution effect on O_2_ and CO_2_ was mathematically compensated for in the analysis stream.^61^ O_2_ consumption and CO_2_ production were measured for each mouse every 5 min for 30 s. Incurrent air reference values were determined every 4 cages. The respiratory exchange rate (RER) was calculated as the ratio of CO_2_ production to O_2_ consumption.

### Bone marrow stromal cell isolation and osteoblast culture

Primary murine bone marrow stromal cells (BMSCs) were isolated as previously described.^62^ Briefly, the distal and proximal ends of the femur, tibia and iliac crests were cut open after removing adherent soft tissue. Total bone marrow, isolated by centrifugation at 12 000 r·min^−1^ for 30 s, was plated in complete α-MEM (α-MEM (Sigma; M0450), 10% FBS (Avantor; 89510-186), 1% penicillin/streptomycin (Sigma; P4333)). These cells were then incubated at 37 °C in the presence of 5% CO_2_. Iliac crests were not used for isolating BMSCs from the control and ΔATGL strains. As per the plastic adherence theory, the nonadherent hematopoietic cells were washed away, whereas the adherent mesenchymal stromal cells adhered to the plastic flask.^63,64^ This adherent population was trypsinized after 48 h, counted and plated in appropriate tissue culture-treated plates at appropriate numbers. BMSCs were then cultured in osteogenic medium (complete α-MEM, 50 μg·mL^−1^ ascorbic acid (Sigma; A4544), and 5 mmol·L^−1^ β-glycerol phosphate (Sigma; G9422)) to induce osteoblast differentiation after the cells became 80% confluent.^62^ For the experiment performed in the absence of osteogenic medium, cells were kept in culture in complete α-MEM medium without ascorbic acid and β-glycerol phosphate.

### Calvarial osteoblast isolation and culture

Calvarial osteoblasts were isolated as previously described.^65^ Briefly, 5-day-old pups were euthanized by quick decapitation, and the calvaria were isolated, cleaned and transferred to sterile PBS. They were then digested in 10 mg·mL^−1^ Collagenase A solution 5 times, keeping them in a 37 °C shaker for 20 minutes for the first two digestions and 30 minutes for the last three. The last three digests were collected, and the cells were cultured in complete α-MEM medium. These cells were trypsinized after 48 h, counted and plated in appropriate tissue culture-treated plates at appropriate numbers and cultured in osteogenic medium.

### Immunostaining, imaging, and analysis

BMSCs were seeded on collagen-treated glass coverslips at a density of 1.75× 10^5^ cells per mL and grown in the presence of osteogenic medium. The cells were fixed at the mentioned time points using neutral-buffered, methanol-free 4% formaldehyde for 20 minutes. For specific experiments, cells were cultured in the presence or absence of 200 μmol·L^−1^ oleic acid (Sigma; O3008) in cell culture medium for 48 h. To investigate the effect of ATGListatin or etomoxir, cells were incubated with 50 μmol·L^−1^ ATGListatin (Signa; SML1075), 10 μmol·L^−1^ etomoxir (Sigma; E1905) or DMSO (vehicle) for 6 h or 3 h, respectively, before fixing and staining at the indicated time points during differentiation. The concentration of ATGListatin used was 50 μmol·L^−1^ for all experiments unless mentioned otherwise. The cells were then washed thrice in 1× phosphate-buffered saline (PBS) and then stained for lipid droplets using 10 μmol·L^−1^ BODIPY 493/503 (Thermo Fisher; D-3922) solution for 1 h at room temperature before being washed three more times with 1× PBS. For immunostaining with Runx2, after fixation, the cells were blocked and permeabilized in 0.2% gelatin B (Sigma; G-9391) with 0.1% saponin (Sigma; 47036) in PBS for 10 minutes. Immunostaining with Runx2 (Cell Signaling Technology; D1L7F) was performed overnight in 0.2% gelatin B with 0.01% saponin at 4 °C. After washing three times with PBS, the cells were stained with secondary antibody (Invitrogen; Alexa Fluor goat anti-rabbit 647; A32733) in the same buffer for 1 h at room temperature. Finally, the cells were washed thrice in PBS and once in distilled water and then mounted on slides. The coverslips were mounted on slides using Prolong Glass Antifade Mountant with NucBlue (Thermo Fisher; P36981). Confocal Z stacks with 0.30 mm thickness were taken using a Zeiss LSM 880. Lipid droplets were identified and counted using a built-in object identification program in Gen5 software. The same program was used to measure the intensity and size of these identified lipid droplets.

### RNA isolation

For BMSC and osteoblast experiments, cells were seeded at a density of 5.0 ×10^5^ per mL, cultured in osteogenic medium in the absence or presence of ATGListatin in some instances, and harvested at the indicated time points during differentiation for total RNA. RNA was isolated from cells either by a kit-based method using the ReliaPrep RNA Cell Miniprep system (Promega; Z6010) following the manufacturer’s protocol for RNA sequencing or by an organic precipitation method following lysis of the cells with TRIzol (Ambion; 15596018) for validation by quantitative real-time PCR. For the RNA sequencing experiment, BMSCs were isolated from male mice. For bone RNA extraction, flash frozen, flushed femur cortex (devoid of marrow elements) was pulverized using a Freezer Mill. Total RNA was isolated using the organic precipitation method following lysis with TRIzol. The precipitated RNA was further cleaned up using a clean-up kit from Qiagen (RNeasy MinElute Cleanup Kit; 74204). For adipose tissue RNA extraction, adipose tissue was lysed in TRIzol using a handheld homogenizer (Fisher; Homogenizer 850). RNA was isolated from the lysed tissue by organic precipitation. Isolated RNA was qualitatively and quantitatively estimated using Nanodrop One (Thermo Fisher) before making downstream applications.

### Next-gen sequencing

The Vanderbilt Technologies for Advanced Genomics (VANTAGE) Core performed QC analysis of the RNA using the Agilent Bioanalyzer and an RNA Qubit assay. RNA-Seq libraries were prepared using 200 ng of total RNA and the NEBNext rRNA Depletion Kit (NEB, Cat: E6310X) per the manufacturer’s instructions. This kit employs an RNaseH-based method to deplete both cytoplasmic (5 S rRNA, 5.8 S rRNA, 18 S rRNA and 28 S rRNA) and mitochondrial ribosomal RNA (12 S rRNA and 16 S rRNA) from human, mouse, and rat total RNA preparations. The mRNA was enriched via poly-A-selection using oligoDT beads, and then the RNA was thermally fragmented and converted to cDNA. The cDNA was adenylated for adaptor ligation and PCR amplification. Sequencing was conducted using an Illumina HiSeq 2500. All samples were indexed, and multiplex sequencing was conducted on the HiSeq to generate the datasets with the customer-specified sequence yield and read length in either single or paired end format. The raw data were submitted to VANGARD for analysis. Adapters were trimmed by Cutadapt (v2.10). After trimming, reads were mapped to the mouse genome GRCm38.p6 using STAR (v2.7.8a) and quantified by feature counts (v2.0.2). DESeq2 (v.1.30.1) was used to assess differential expression between two groups. WebGestaltR (v0.4.4) was used to perform functional enrichment analysis against the Gene Ontology and KEGG databases. GSEA (v4.2.3) was used to find enriched pathways against hallmark gene sets in MSigDB (v7.5.1).

### qRT‒PCR analysis

Complementary DNA (cDNA; 12.5 ng) was prepared from purified RNA (Thermo Fisher; 4374967) and utilized for downstream applications. Expression analysis of selected genes by quantitative real-time PCR (qRT‒PCR) with SYBR green (Thermo Fisher; PowerUP SYBR Green; A25742) on a QuantStudio 5 (Thermo Fisher) with the following conditions: 2 min at 50 °C, 10 min at 95 °C, (15 s at 95 °C, 1 min at 60 °C) × 40 cycles followed by dissociation curve analysis (15 s at 95 °C, 1 min at 60 °C, 15 s at 95 °C). Analysis was performed by normalizing the expression of the target gene to the housekeeping gene *Hprt* for BMSCs and nonbone tissues and *B2m* for bone expression within the same sample to determine ΔCt. The ΔCt was transformed (2^−ΔCt^). For determining normalized to control/DMSO expression of target genes (2^−ΔCt^) with treatment/average of (2^−ΔCt^) in control/DMSO group” was determined. The sequences of primers used are listed in Table S3. To validate primer efficiencies, a concentration gradient of cDNA was used as a template.

### Fluorescent fatty acid pulse chase for measuring cellular lipolysis

Cells seeded at a density of 5×10^5^ per mL were incubated with complete α-MEM containing 2 μmol·L^−1^ BODIPY 558/568 C_12_ (Thermo Fisher; D3835) for 16 h at the indicated time points during osteoblastogenesis. The cells were then washed three times with 1× PBS and chased for 6 h in α-MEM medium with 2% fatty acid-free BSA (Sigma; A8806) and 1% penicillin/streptomycin 10 μmol·L^−1^ Triacsin C (fatty acyl CoA synthetase inhibitor, Sigma; T4540) in the presence or absence of ATGListatin. The cells were washed twice in 1× PBS and lysed in 1% Triton X-100 for lipid extraction. Lipids were extracted following the Bligh and Dyer method.^66^ Briefly, four volumes of chloroform:methanol (1:2) (Sigma; 319988, 179337) were added to the lysate, and the mixture was vortexed. One volume of 50 mmol·L^−1^ citric acid (Sigma; 251275), one volume of distilled water and one volume of chloroform were added sequentially and again vortexed. This final mixture was then centrifuged at 10 000 r·min^−1^ for 10 minutes. The lower organic phase was isolated and dried. The dried lipid extract was resuspended in a chloroform:methanol (2:1) mixture and loaded on TLC (Sigma; 1.05553.0001). Lipids were separated by developing TLC at room temperature in a solvent system of cyclohexane:ethyl acetate (1:2)^67^ (Sigma; 34855,650528) along with a triglyceride standard (Avanti Polar Lipids; 810272 P), and fluorescent lipids were visualized using iBright 1500 (Thermo Fisher). Fiji was used to analyze the fluorescent lipid bands, and the integrated density of the lipid bands from each sample was normalized to the integrated density for the origin of that sample.

### Osteoblast von Kossa staining

Osteoblasts differentiated in the presence or absence of ATGListatin during the indicated time frame were assessed for mineralization by Von Kossa (VK) staining 10 days after ex vivo differentiation. Briefly, cells were washed three times in 1× PBS and then fixed with 10% neutral-buffered formalin (NBF) for 15 minutes at 37 °C. The fixed cells were then incubated in 5% silver nitrate in the presence of UV for 1 h. After 1 h, the cells were washed with distilled water and incubated with 5% sodium thiosulfate for 3 minutes. The cells were washed three times in distilled water, dried and imaged.

### Fluorescent fatty acid pulse chase for investigating mitochondrial localization

BMSCs were seeded on collagen-treated glass coverslips at a density of 1.75× 10^5^ per mL and grown in the presence of osteogenic medium. The cells were incubated with complete α-MEM containing 2 μmol·L^−1^ BODIPY 558/568 C_12_ (Thermo Fisher; D3835) for 16 h at the indicated time points during osteoblastogenesis. The cells were then washed three times with 1× PBS and chased for 6 h in complete medium with or without ATGListatin. After 6 h, the cells were washed and stained with 100 nmol·L^−1^ MitoTracker Deep Red FM (M22426) in incomplete medium for 30 minutes, washed with PBS, fixed in neutral-buffered, methanol-free 4% formaldehyde for 20 minutes and finally mounted on slides with Prolong Glass Antifade Mountant with NucBlue (Thermo Fisher; P36981). Confocal Z stacks with 0.30 mm thickness were taken using a Zeiss LSM 880. Colocalization analysis was performed on a per cell basis from a single stack with maximum mitochondria using ImageJ to calculate Mander’s coefficient using the threshold (tM2), where channel 1 was far red (644/665) for Mito Tracker Deep Red, denoting mitochondria, and channel 2 was red (558/568) for C_12_ fatty acids.

### ATP rate assay

To measure ATP produced by either glycolysis or oxidative phosphorylation in real time with a Seahorse ATP rate assay, BMSCs were plated in Seahorse XFe 96-well plates at 2.5 × 10^4^ cells per well and cultured under osteogenic conditions. At specified timepoints, ATP rate assays were performed (Agilent; 103592-100). Briefly, after removing the cell culture medium, the cells were washed with either basal assay DMEM (Agilent; 103575-100), and then the assay was performed in the absence or presence of 1, 5 or 10 mmol·L^−1^ glucose (Agilent; 103577-100), 2 mmol·L^−1^ glutamine (Agilent; 103579-100), 1 mmol·L^−1^ sodium pyruvate (103578-100), 200 nmol·L^−1^ insulin (Sigma; I9278), 60 μmol·L^−1^ oleic acid-BSA (Sigma; O3008), or 0.5 mmol·L^−1^ carnitine or in the presence of only 100 μmol·L^−1^ oleic acid in that same medium. For specific experiments, cells were cultured in the presence of 200 μmol·L^−1^ oleic acid in cell culture medium for 48 h. Subsequently, a final concentration of 2 μmol·L^−1^ oligomycin (Sigma; 75351) and 1 μmol·L^−1^ rotenone/1 μmol·L^−1^ antimycin A (Sigma; R8875, A8674) were injected during assays through port A and port B, while oxygen consumption rates (OCRs) and extracellular acidification rates (ECARs) were monitored in real time. For specific experiments, cells were incubated with 10 μmol·L^−1^ etomoxir or 5 μmol·L^−1^ UK5099 (Sigma; PZ0160) for 1 h in the assay medium before starting the assay or with or without ATGListatin for 24 h in cell culture medium or with both ATGListatin for 24 h in cell culture medium and etomoxir for 1 h in assay medium before the assay. Under both conditions, the assays were performed in the presence of the inhibitors in the assay medium. Considering the stoichiometry of the glycolytic pathway, the percentage of glycolysis was based mainly on the ECAR. Conversely, the rate of oxygen consumption that is coupled to ATP production during oxidative phosphorylation was calculated as the OCR that was inhibited by addition of the ATP synthase inhibitor oligomycin. This assay measured the flux of both H^+^ production, as indicated in ECAR, and O_2_ consumption, reported as OCR, simultaneously. By obtaining these data under basal conditions and after serial addition of mitochondrial inhibitors (oligomycin and rotenone/antimycin A), the rate of glycolysis and oxidative phosphorylation were able to be measured. Hoechst 33342 stain (Thermo Scientific; 62249) was also injected in the last port, and a Cytation 5 (BioTek) was used to provide cell counts, both for normalization and to monitor proliferation throughout differentiation.

### Dual-energy X-ray absorptiometry

Dual-energy X-ray absorptiometry (DXA) was performed on the control and ΔATGL mice 8 weeks after starting the purified diet before their sacrifice in the prone position by using a Faxitron UltraFocus (Hologic). The instrument was calibrated for offset images, dark images, and flat-field images before the measurement by a method provided by the manufacturer.

### Micro-computed tomography (μCT)

Tibiae from the mice were cleaned of soft adhering tissue, placed in 10% neutral-buffered formalin (NBF) for 48 h, and then stored in 70% ethanol. The proximal and mid-diaphyses of these tibiae were imaged using an ex vivo micro-computed tomography (μCT) scanner (μCT 50, Scanco Medical AG, Brüttisellen, Switzerland). With a peak X-ray tube intensity and current of 70 kVp and 114 mA, respectively, 500 projections per full rotation of the sample, and an integration time of 300 ms, image stacks with an isotropic voxel size of 6 μm were acquired for the tibia metaphysis and diaphysis (310 slices each). Trabecular bone was analyzed by identifying a region of interest (ROI) 180 μm distal from the proximal tibia growth plate to include a region of secondary spongiosa extending distally 1.2 mm. Cortical bone was analyzed to include 1.2 mm ending at the tibiofibular junction.

### Lipid isolation from bone and TLC analysis

The flushed tibia cortex (devoid of marrow elements) was pulverized and used to harvest total lipid by the Bligh and Dyer method^66^ mentioned previously. However, the powder obtained from the total flushed tibia after pulverization was weighed before adding a chloroform:methanol (1:2) mixture for normalization. Additionally, the powder was kept overnight in chloroform:methanol mixtures in a 37 °C water bath for better and complete extraction before the following steps were performed. Finally, the dried lipid extract was resuspended in an equal volume of (80 μL) chloroform: methanol (2:1) mixture and loaded on TLC (Sigma; 1.05553.0001). Lipids were separated by developing TLC at 4 °C in a solvent system of hexanes:diethyl ether:acetic acid (70:30:1) (Sigma; 293253, Emparta; 1.07026.2500, Sigma; 695092). The TLCs were stained using 10% (wt/vol) copper sulfate in an 8% (vol/vol) phosphoric acid solution and then charred at 120 °C for visualization of lipids. Fiji was used to analyze the lipid bands, and the integrated density of the lipid bands from each sample was normalized to the weight of the powdered bone sample.

### Protein isolation and Western blot analysis

For BMSC and osteoblast experiments, cells were seeded at a density of 5.0 ×10^5^ per mL, cultured in osteogenic medium in the absence or presence of ATGListatin in some instances, and harvested at the indicated time points during differentiation for total protein. Protein was isolated from cells by lysing the cells with 1× RIPA (Cell Signaling Technology; 9806) buffer in the presence of protease inhibitor cocktail (Roche; 04693116001) and phosphatase inhibitor cocktail (Roche; 04906845001). For bone protein extraction, flash-frozen, flushed femur cortex (devoid of marrow elements) was pulverized using a Freezer Mill. Total RNA was isolated following lysis with 1× RIPA buffer in the presence of protease inhibitor and phosphatase inhibitor cocktail. Protein estimation was performed using the BCA method. Equal amounts of protein (15-35 µg) from each sample were loaded and resolved using sodium dodecyl sulfate‒polyacrylamide gel electrophoresis (SDS‒PAGE), followed by transfer to PVDF membranes (Bio-Rad; 1704156). The membrane was incubated with no-stain protein labeling reagent (Invitrogen; A44449) for normalization and assessment of equal loading and finally probed for specific proteins ATGL (Cell Signaling Technology; 2439), AMPKα (Cell Signaling Technology; 5831) or phospho-AMPKα (Thr172; Cell Signaling Technology; 2535) antibodies.

### Histology

The eWAT (epididymal white adipose tissue) collected from the control and knockout mice was fixed in 10% neutral-buffered formalin (NBF) for 48 h and then stored in 70% ethanol. These samples were submitted to the Translational Pathology Shared Resource (TPSR) core for further processing. Briefly, these samples were subjected to sequential ethanol dehydration followed by embedding in paraffin. The embedded tissues were then sectioned on glass slides at 5 µm thickness, deparaffinized, and subjected to H & E staining. The number and volume of the adipocytes present in the tissue section was further quantified post microscopy imaging using BioQuant® Osteo 2018 version 18.2.6 (BioQuant® Image Analysis Corporation, Nashville, TN).

### Bone histomorphometry

Dynamic bone formation was assessed by prior injection of sequential doses of calcein (10 mg·kg^−1^ body weight) and alizarin (1,2-dihydroxyanthraquinone) (30 mg·kg^−1^ body weight) 7 and 2 days prior to sacrifice, respectively (5-day interval). and embedded in methylmethacrylate (MMA). Briefly, the cleaned tibias were subjected to sequential acetone dehydration, and the dehydrated tibias were infiltrated using a mixture of destabilized methlymethacrylate (90%), dibutylphthalate (10%) and benzoyl peroxide (0.05%) for 3 days at 4 °C. The infiltrated bones were embedded in embedding solution, which was a mixture of the same chemicals with different concentrations of the abovementioned chemicals (85%, 15% and 4%) for 3-4 days in a 37 °C incubator. The embedding was done by pouring the embedding solution on top of base made up of 5% benzoyl peroxide in infiltration solution before the bones were placed in it. Then, the bones were embedded in MMA plastic, sectioned (5 µm) on a transverse plane and subjected to downstream analysis. Single or double labels were measured to quantify MS/BS and BFR/BS. The bone sections were stained with trichrome stain. The number of osteoblasts and osteoclasts per bone perimeter were measured at standardized sites under the growth plate at a magnification of 20 ×. Osteoblasts were identified as plump cuboidal cells lining the trabecular bone surface ( > 3 tough cells), osteoclasts were identified as large, multinucleated cells on the bone surface (mostly near the eroded surface), and bone marrow adipocytes were identified as white ‘ghost cells’ within the marrow area excluding vasculature and/or sectioning artifacts. Analyses were performed using BioQuant® Osteo 2018 version 18.2.6 (BioQuant® Image Analysis Corporation, Nashville, TN). All parameters and regions of analysis are reported as per the guidelines of the nomenclature committee of the American Society of Bone and Mineral Research (ASBMR).^68,69^

### Serum lipid analyses

Blood was collected from the carotid arteries of mice immediately before termination. Serum was isolated by allowing blood to clot for 30 min at room temperature, followed by centrifugation at 4 °C at 3 000 r·min^−1^ for 10 min. Serum was submitted to Vanderbilt Analytical Service Core (VASC) for further lipid analysis. Briefly, triglycerides and cholesterol were measured by standard enzymatic assays. FFAs were measured with a commercially available enzymatic kit from Fujifilm Healthcare Solutions (HR Series NEFA-HR).

### Fasting blood glucose

Mice were fasted for 6 h prior to testing. Following this period, blood was collected via a tail nick, and blood glucose was determined using an AlphaTRAK2 glucometer.

### Transmission electron microscopy

Freshly isolated tibiae were fixed in 2.5% glutaraldehyde/2% paraformaldehyde for 48 h and then decalcified (Thermo Scientific; 8340-1). The tibiae were then washed 3 times for 5 min each in 0.1 mol·L^−1^ cacodylate buffer before being subjected to sequential postfixation in 1% tannic acid and then 1% osmium tetroxide and en bloc staining with 1% uranyl acetate for 60 min each. The samples were dehydrated by passing through a graded ethanol series. Tissues were infiltrated with Epon-812 using propylene oxide as the transition solvent and polymerized at 60 °C for 48 h with resin for five days. Thin sections (70 nm) were cut using a Leica UC7 and a Diatome diamond knife. Thin sections were positioned on grids and stained with uranyl acetate and Reynold’s lead citrate. These sections were then imaged on a Tecani T12 TEM operating at 100 keV using an AMT CMOS camera.

### Statistical analysis

Statistical analyses were performed in GraphPad Prism V9. The normal distribution assumption for sample size < 40 was evaluated using the Shapiro‒Wilk normality test. Statistically significant differences across two groups were evaluated using Student’s two-tailed unpaired *t* test with a significance defined as *P* < 0.05 when normal distribution was met; when normality assumptions were not met, an alternative nonparametric test (Mann‒Whitney test) was used. The data are expressed are either the mean ± standard deviation (SD) or the mean ± standard error of mean (SEM) as described. Micro-CT analysis and RNA sequencing were performed with 5 mice from each group. For cell culture experiments, stromal cells were obtained and pooled from 6 mice in each group. For the statistical analysis of RNA sequencing, see the “RNA isolation” and “Next-gen sequencing” sections above.

### Supplementary information

Supplemental Material

Table S2
