# Supplementary material for: Lipolysis supports bone formation by providing osteoblasts with endogenous fatty acid substrates to maintain bioenergetic status
Source: Bone Res. 2023 Nov 24;11:62. doi: 10.1038/s41413-023-00297-2 (PMC10673934; doi:10.1038/s41413-023-00297-2)
Supplement: Supplementary file 1 — Supplemental Material [file 41413_2023_297_MOESM1_ESM.docx]

**Table S1.**

| **Sample** | **Input reads** | **Uniquely mapped reads** | **Mapped to multiple loci** | **Mapped to too many loci** | **Uniquely mapped ratio** | **Mapped ratio** |
| --- | --- | --- | --- | --- | --- | --- |
| Day0_S1 | 38937965 | 36923811 | 1123840 | 39584 | 0.9482727 | 0.977135 |
| Day0_S2 | 43384838 | 41276763 | 1294534 | 45232 | 0.9514099 | 0.981248 |
| Day7_S1 | 40471534 | 38256780 | 1165070 | 39249 | 0.9452763 | 0.974064 |
| Day7_S2 | 39680635 | 37548065 | 1314926 | 52356 | 0.9462567 | 0.979394 |
| Day7_S3 | 57473138 | 54448484 | 1814423 | 81176 | 0.9473727 | 0.978943 |

**Table S3.**

| **Primer Name** | **Primer Sequence** | **Primer Efficiency** |
| --- | --- | --- |
| Runx2_F | CGACAGTCCCAACTTCCTGT | -3.3 |
| Runx2_R | CGGTAACCACAGTCCCATCT |  |
| Pnpla2_F | GACGGAGAGAACGTCATCATATC | -3.3 |
| Pnpla2_R | CCACAGTACACCGGGATAAAT |  |
| Sp7_F | GAAGTTCACCTGCCTGCTCTGT |  |
| Sp7_R | CGTGGGTGCGCTGATGT |  |
| Alpl_F | GGTATGGGCGTCTCCACAGT |  |
| Alpl_R | GCCCGTGTTGTGGTGTAGCT |  |
| Col1a1_F | CGTCTGGTTTGGAGAGAGCAT |  |
| Col1a1_R | GGTCAGCTGGATAGCGACATC |  |
| Sparc_F | TGACCTAGACAACGACAAGTACATTG |  |
| Sparc_R | TGCTCCTTGATGCCAAAGC |  |
| Bglap2_F | TGAGCTTAACCCTGCTTGTGACGA |  |
| Bglap2_R | AGGGCAGCACAGGTCCTAAATAGT |  |
| Cpt1a_F | CAAAGATCAATCGGACCCTAGAC |  |
| Cpt1a_R | CGCCACTCACGATGTTCTTC |  |
| Pfkb1_F | TGAGCTGCCCTATCTCAAGTGT |  |
| Pfkb1_R | TCCACTCTGCAGCCATAAGC |  |
| Acaca_F | GGCAGCTCTGGAGGTGTATG |  |
| Acaca_R | TCCTTAAGCTGGCGGTGTT |  |
| Fasn_F | GCTGCGGAAACTTCAGGAAAT |  |
| Fasn_R | AGAGACGTGTCACTCCTGGACTT |  |
| Cd36_F | GGAACTGTGGGCTCATTGC |  |
| Cd36_R | CATGAGAATGCCTCCAAACAC |  |
| Fabp5_F | GCCAAGCCAGACTGTATCATTA |  |
| Fabp5_R | CTCCCAGGTTACAAGAGAACAC |  |
| Lipe_F | CATCAACCACTGTGAGGGTAAG |  |
| Lipe_R | AAGGGAGGTGAGATGGTAACT |  |
| Lipa_F | GAGTTCTGGGCCTTCAGTTT |  |
| Lipa_R | GCCTTGAGAATGACCCACATA |  |
| Hprt_F | GCCTAAGATGAGCGCAAGTTG |  |
| Hprt_R | TACTAGGCAGATGGCCACAGG |  |
| Pnpla2_Genotyping_F | GAGTGCAGTGTCCTTCACCA |  |
| Pnpla2_Genotyping_R | ATCAGGCAGCCACTCCAAC |  |
| Prx1-Cre_Genotyping_F | TTCCCGCAGAACCTGAAGATG |  |
| Prx1-Cre_Genotyping_R | CCCCAGAATGCCAGATTACG |  |

**Supplementary Figures**


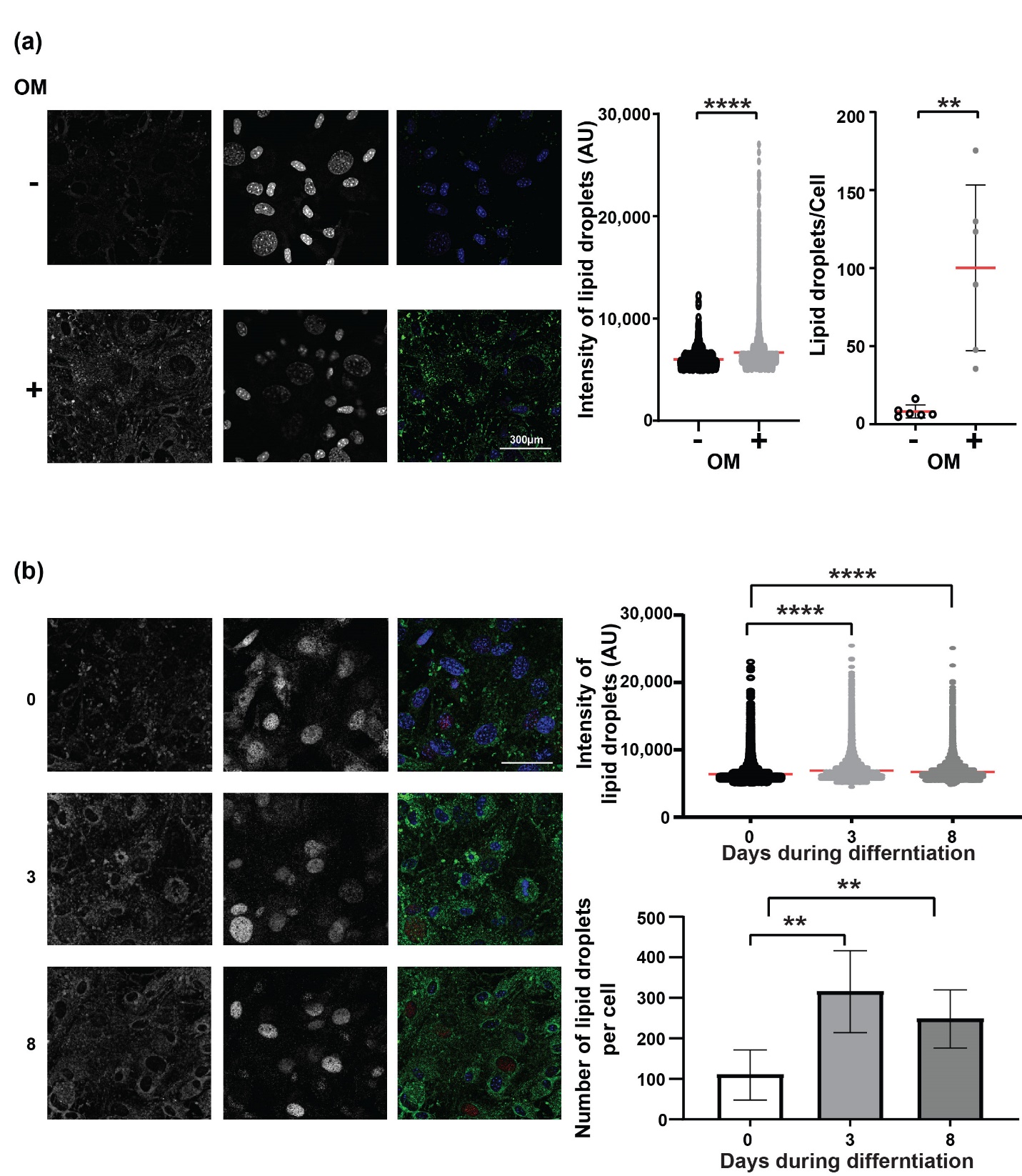


**Figure S1.** (a) Representative confocal image of stromal cells kept in absence (-) or presence (+) of osteogenic media (OM) for 8 days. Cells were immunostained for Runx2 (red in merged panel) along with mounting with DAPI (blue in merged panel). Cellular lipid droplets were stained with BODIPY 493/503 (green in merged panel). Panel 1 and 2 show monochrome images of lipid droplet and Runx2 stained nuclei staining respectively whereas panel 3 shows merged image. Quantification of intensity of BODIPY 493/503 stained lipid droplet in cells without (-) (open circle) and with (+) (closed gray circle) osteogenic media where each dot represents intensity of one lipid droplet. Data are mean ± standard error of mean where intensity of each lipid droplets were measured from independent images captured in 6 different field of view of the coverslip (*n*=6) with BMSCs obtained from 4, 9-week-old mice.t tests were done assuming normal distribution since, data points were more than 40, to determine significance between two groups where *,p <0.05, **,p <0.01, ***,p <0.001, ****,p <0.0001. Quantification of number of lipid droplets per cell in cells without (-) (open circle) and with (+) (closed gray circle) osteogenic media. Data are mean ± standard deviation (SD) where lipid droplets per cell were counted from independent images captured in 6 different field of view (n=6) for each experiment from pooled BMSCs obtained from 4, 9-week-old mice. Number of lipid droplets counted from each image were divided by number of cells (number of DAPI positive nucleus) in that image to get lipid droplets per cell. t tests or non-parametric Mann-Whitney tests were done accordingly after testing normal distribution using Shapiro-Wilk normality test to determine significance between two groups where *, p <0.05, **, p <0.01, ***, p <0.001, ****, p <0.0001. OM= Osteogenic Media. (b) Representative confocal image of calvarial osteoblasts kept in presence of osteogenic media for 8 days at indicated time points. Cells were immunostained for Runx2 (red in merged panel) along with mounting with DAPI (blue in merged panel). Cellular lipid droplets were stained with BODIPY 493/503 (green in merged panel). Panel 1 and 2 show monochrome images of lipid droplet and Runx2 stained nuclei staining respectively whereas panel 3 shows merged image. Quantification of intensity of BODIPY 493/503 stained lipid droplet in cells at indicated time points in osteogenic media. Data are mean ± standard error of mean where intensity of each lipid droplets were measured from independent images captured in 6 different field of view of the coverslip (n=6) with pooled BMSCs obtained from 5, 4 day old pups.t tests were done assuming normal distribution since, data points were more than 40, to determine significance between two groups where *,p <0.05, **,p <0.01, ***,p <0.001, ****,p <0.0001. Quantification of number of lipid droplets per cell in cells at indicated time points in osteogenic media. Data are mean ± standard deviation (SD) where lipid droplets per cell were counted from independent images captured in 6 different field of view (*n*=6) for each experiment from pooled BMSCs obtained from 5, 4-day old pups. Number of lipid droplets counted from each image were divided by number of cells (number of DAPI positive nucleus) in that image to get lipid droplets per cell. Paired T-tests or non-parametric Mann-Whitney tests were done accordingly after testing normal distribution using Shapiro-Wilk normality test to determine significance between two groups where *, p <0.05, **, p <0.01, ***, p <0.001, ****, p <0.0001.

**
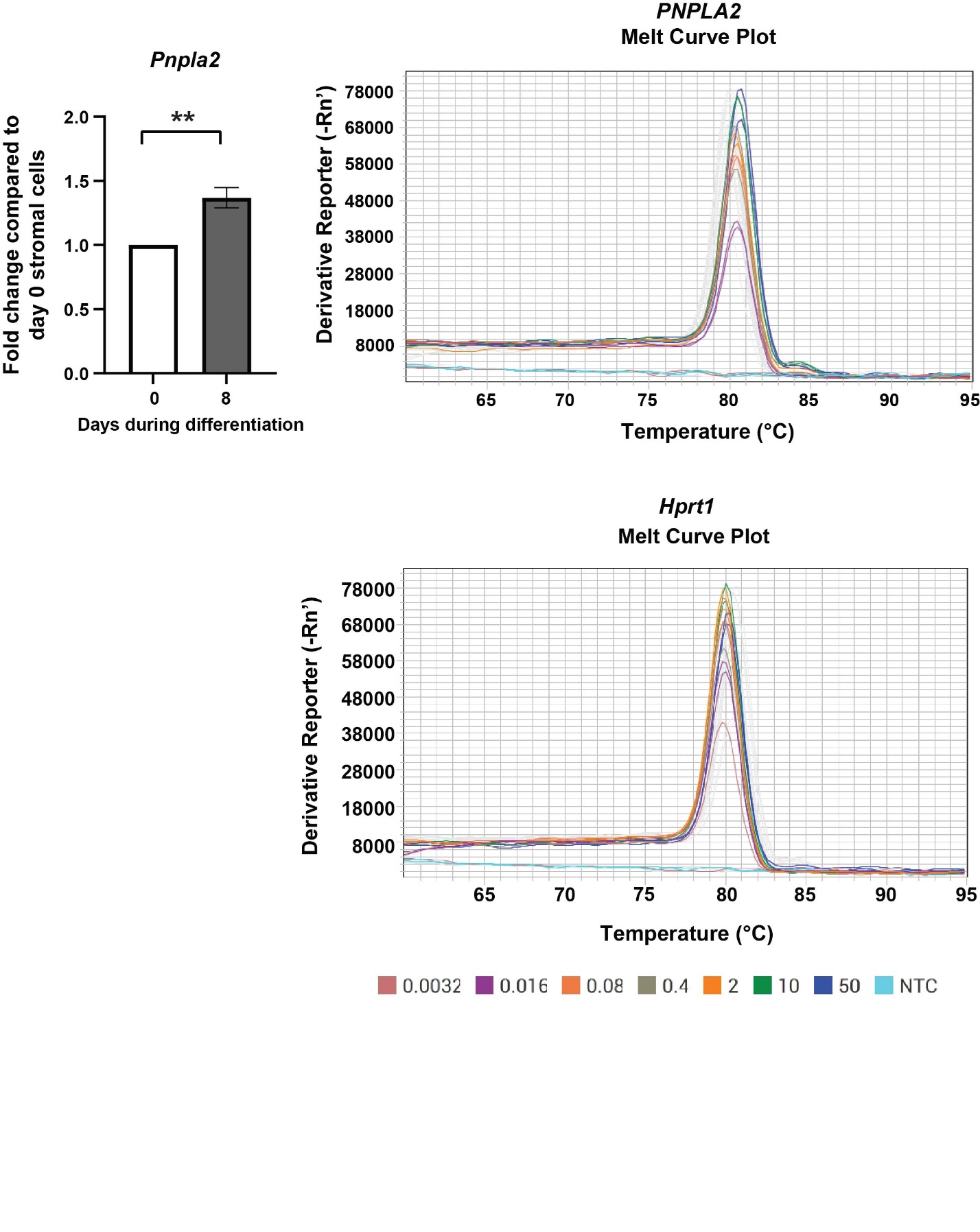
Figure S2.** Quantitative real time PCR derived normalized expression of *Pnpla2* on 8^th^ day of differentiation (gray bar) compared to 0^th^ day stromal cells (open bar). t tests were done after testing normal distribution using Shapiro-Wilk normality test to determine significance between two groups where *, p <0.05, **, p <0.01, ***, p <0.001, ****, p <0.0001. Melt curves for the primer sets used for quantitative real time PCR analysis of *Pnpla2* and housekeeping gene *Hprt1.*


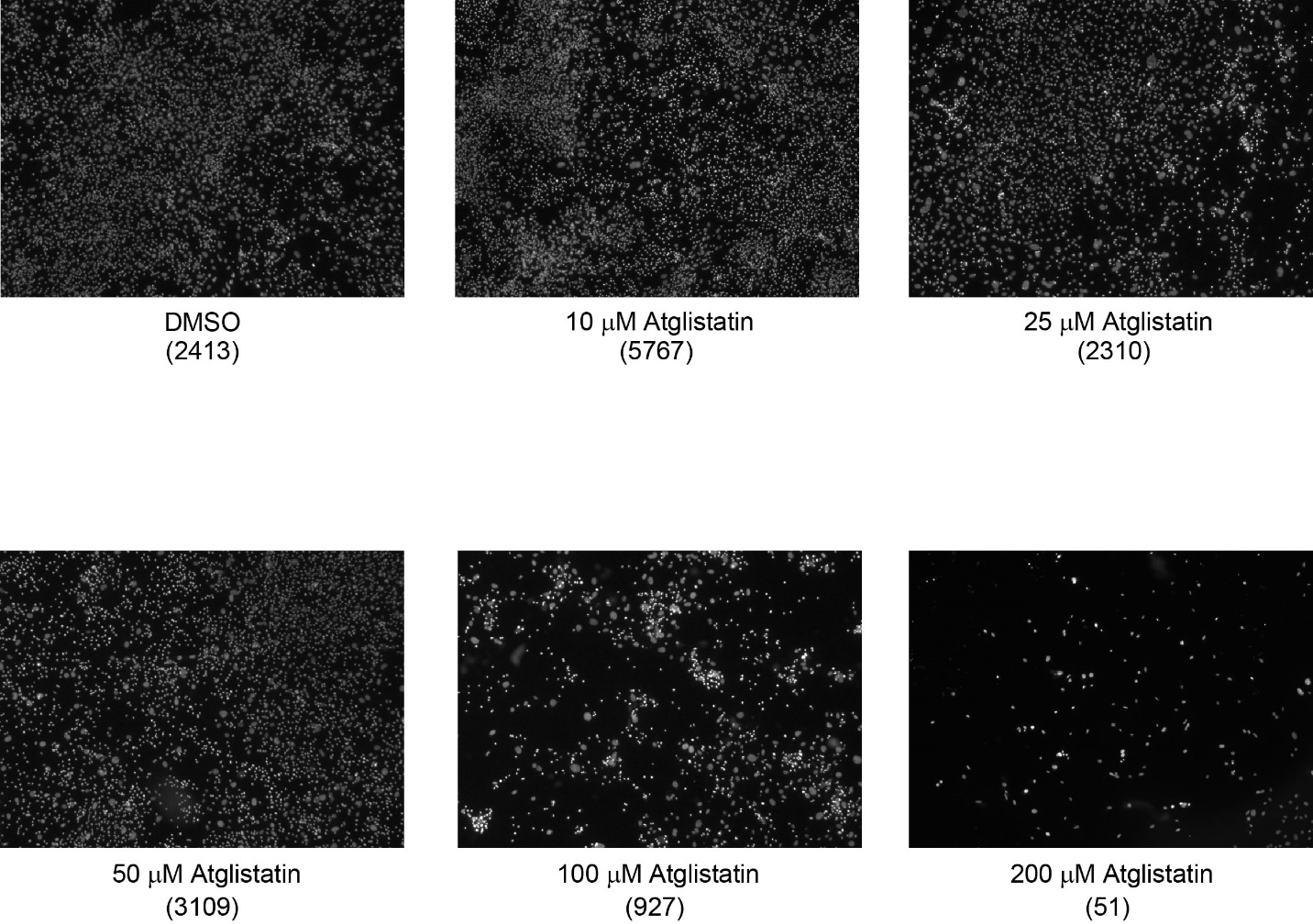


**Figure S3. Dose Curve for ATGListatin Treatment**. Representative images of matured osteoblasts treated with increasing concentration of ATGListatin stained with DAPI. Number mentioned below each image shows number of nuclei present in the field.


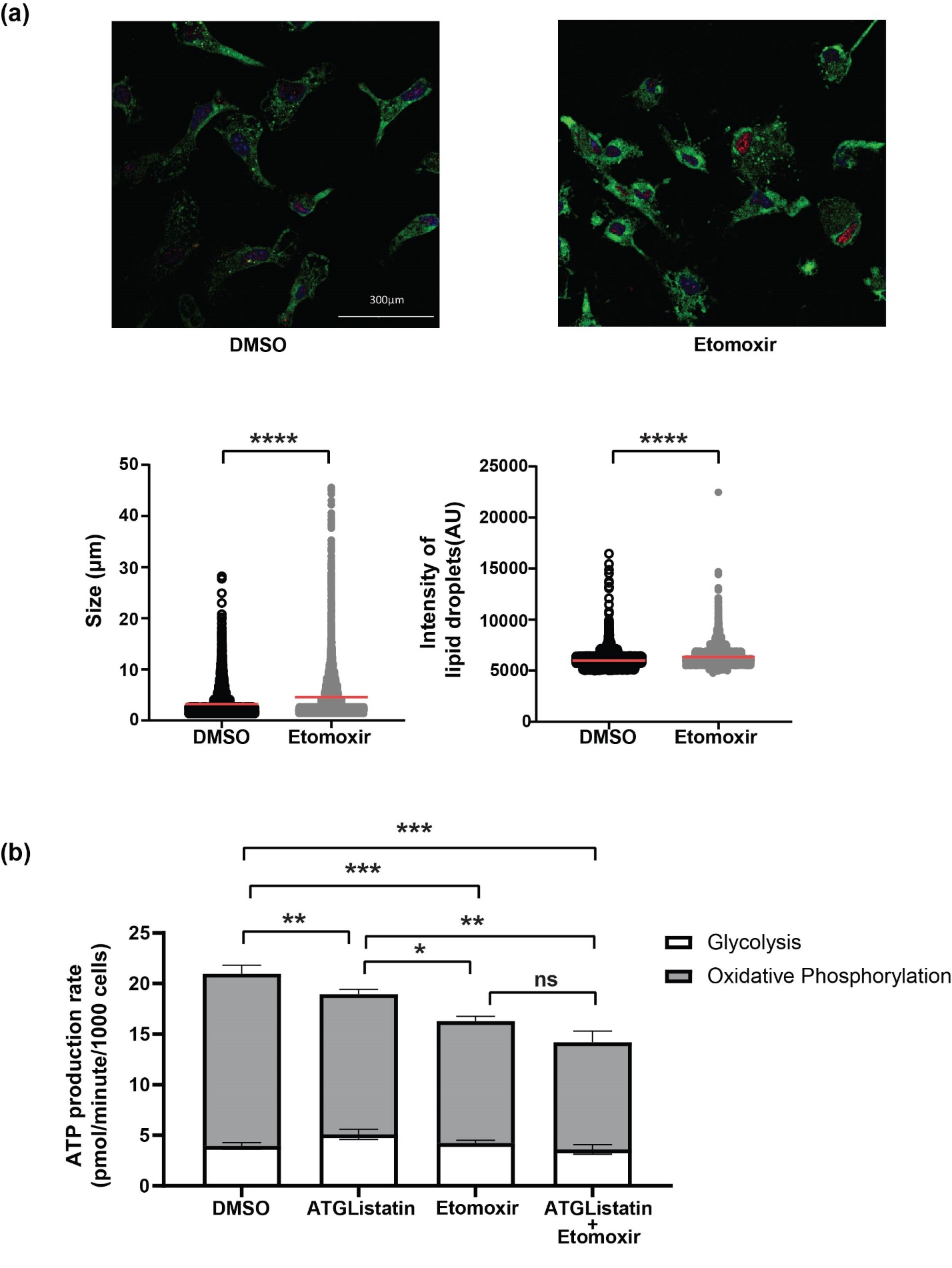


**Figure S4. Effect of blocking β-oxidation on lipid metabolism in osteoblast.** (a) Representative confocal image of *ex vivo* differentiated osteoblasts kept in absence (DMSO) or presence of etomoxir for 3 hours. Cells were immunostained for Runx2 (red) along with mounting with DAPI (blue). Cellular lipid droplets were stained with BODIPY 493/503 (green). Quantification of size and intensity of BODIPY 493/503 stained lipid droplet in cells without (open circle) and with (closed gray circle) etomoxir where each dot represents size or intensity of one lipid droplet. Data are mean ± standard error of mean where intensity of each lipid droplets were measured from independent images captured in 6 different field of view of the coverslip (n=6) with BMSCs obtained from 4, 9-week-old mice. Paired T-tests were done assuming normal distribution since, data points were more than 40, to determine significance between two groups where *,p <0.05, **,p <0.01, ***,p <0.001, ****,p <0.0001. (b) ATP produced by glycolysis (white) versus oxidative phosphorylation (shaded) measured by Seahorse ATP rate assay in absence of any external nutrients in differentiated matrix secreting osteoblasts on 8^th^ day of *ex vivo* differentiation in absence or presence of ATGListatin, Etomoxir (Eto) or both. Data are mean ± standard error of mean of data normalized to cell counts per well with data from minimum 8 wells per group (*n*=8). Paired T-tests or non-parametric Mann-Whitney tests were done accordingly after testing normal distribution using Shapiro-Wilk normality test to determine significance between two groups where *, p <0.05, **, p <0.01, ***, p <0.001, ****, p <0.0001.


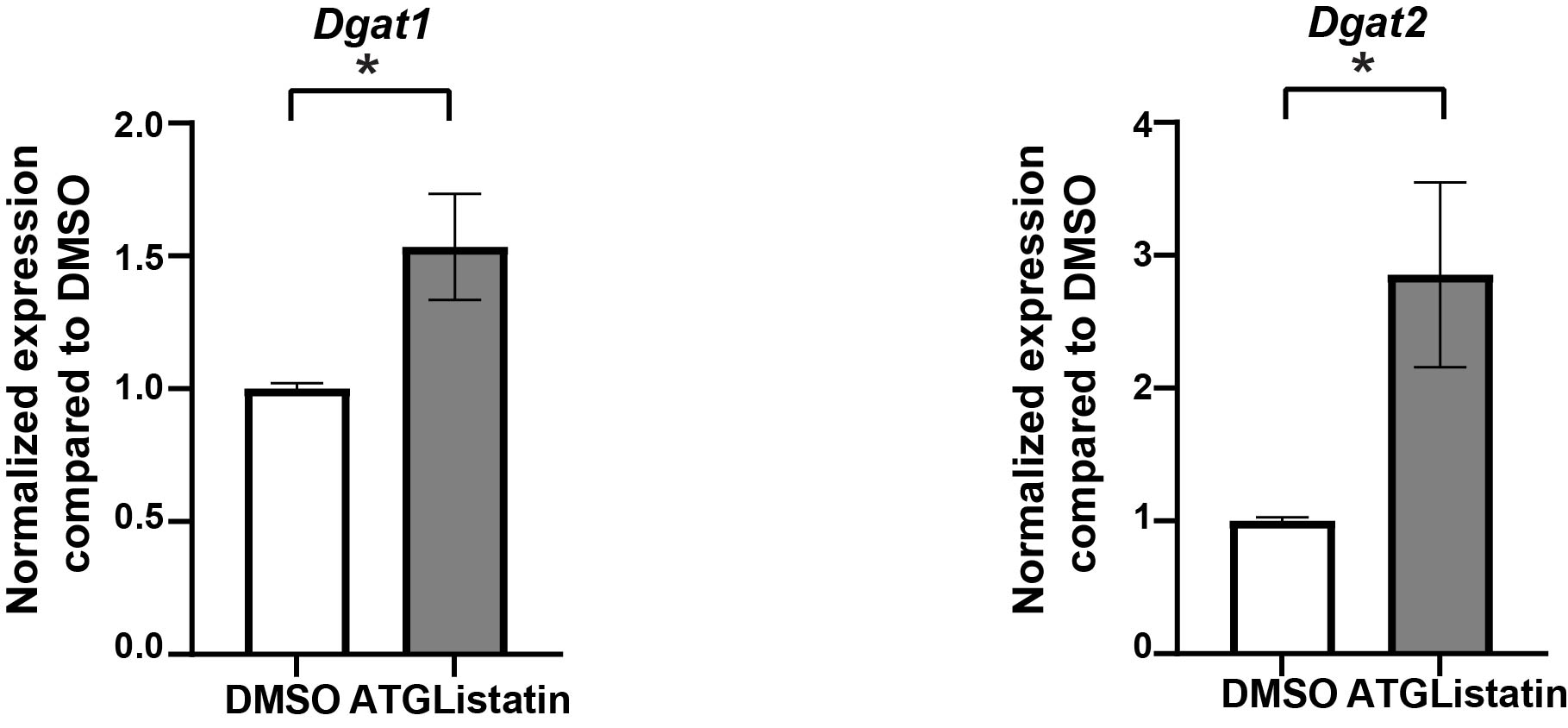


**Figure S5.** Quantitative real time PCR based expression of *de novo* triglyceride genes in presence of DMSO (open bar) or ATGListatin (gray bar) in 10^th^ day differentiated osteoblasts where ATGListatin was present throughout in the ATGListatin treated group. Fold change compared to DMSO was calculated by normalizing the house keeping gene normalized expression of individual genes in the treated group to the mean of housekeeping gene normalized expression of that gene in the DMSO control group. Data are mean ± standard error of mean pooled from 3 independent experiments (N=3) each having 3 technical replicates (*n*=9). t tests or non-parametric Mann-Whitney tests were done accordingly after testing normal distribution using Shapiro-Wilk normality test to determine significance between two groups where *, p <0.05, **, p <0.01, ***, p <0.001, ****, p <0.0001.


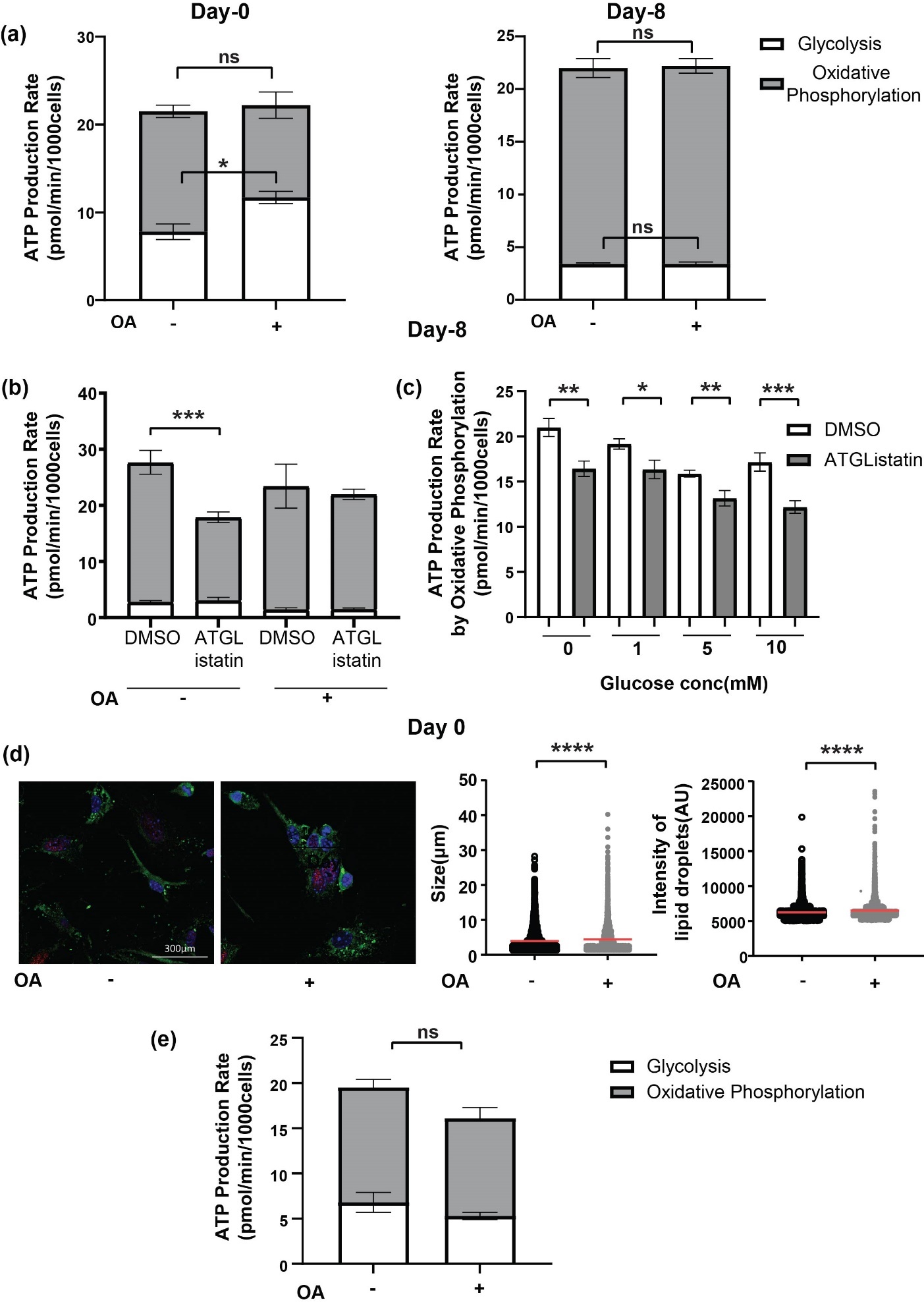


**Figure S6. Effect of presence of exogenous nutrients on lipid metabolism of osteoblasts in presence or absence of ATGListatin.** ATP produced by glycolysis (white) versus oxidative phosphorylation (shaded) measured by Seahorse ATP rate assay in (a) presence or absence of exogenous fatty acid/oleic acid (OA) in BMSCs on 0^th^ day or in differentiated matrix secreting osteoblasts on 8^th^ day of *ex vivo* differentiation, (b) presence or absence of exogenous fatty acid in DMSO or ATGListatin treated differentiated matrix secreting osteoblasts on 8^th^ day of *ex vivo* differentiation. Data are mean ± standard error of mean of data normalized to cell counts per well with data from minimum 6 wells per group (*n*=6). t tests or non-parametric Mann-Whitney tests were done accordingly after testing normal distribution using Shapiro-Wilk normality test to determine significance between two groups where *, p <0.05, **, p <0.01, ***, p <0.001, ****, p <0.0001. (c) ATP produced by oxidative phosphorylation measured by Seahorse ATP rate assay in absence (open bar) or presence (gray bar) of ATGListatin in absence or presence of increasing concentration of exogenous glucose. ATGListatin treated differentiated matrix secreting osteoblasts on 8^th^ day of *ex vivo* differentiation. Data are mean ± standard error of mean of data normalized to cell counts per well with data from minimum 10 wells per group (*n*=10). Paired T-tests or non-parametric Mann-Whitney tests were done accordingly after testing normal distribution using Shapiro-Wilk normality test to determine significance between two groups where *, p <0.05, **, p <0.01, ***, p <0.001, ****, p <0.0001. (d) Representative confocal image of BMSCs cultured in absence or presence of fatty acid/oleic acid (OA) in cell culture media for 48 hours. Cells were immunostained for Runx2 (red) along with mounting with DAPI (blue). Cellular lipid droplets were stained with BODIPY 493/503 (green). Quantification of size and intensity of BODIPY 493/503 stained lipid droplet in cells without (open circle) and with (closed gray circle) oleic acid where each dot represents size or intensity of one lipid droplet. Data are mean ± standard error of mean where intensity of each lipid droplets were measured from independent images captured in 6 different field of view of the coverslip (*n*=6) with BMSCs obtained from 4, 9-week-old mice. Paired T-tests were performed assuming normal distribution since, data points were more than 40, to determine significance between two groups where *,p <0.05, **,p <0.01, ***,p <0.001, ****,p <0.0001. (e) ATP produced by glycolysis (white) versus oxidative phosphorylation (shaded) measured by Seahorse ATP rate assay in absence of any external nutrients in BMSCs cultured in presence or absence of oleic acid in cell culture media for 48 hours. Data are mean ± standard error of mean of data normalized to cell counts per well with data from minimum 7 wells per group (n=7). t tests or non-parametric Mann-Whitney tests were done accordingly after testing normal distribution using Shapiro-Wilk normality test to determine significance between two groups where *, p <0.05, **, p <0.01, ***, p <0.001, ****, p <0.0001.


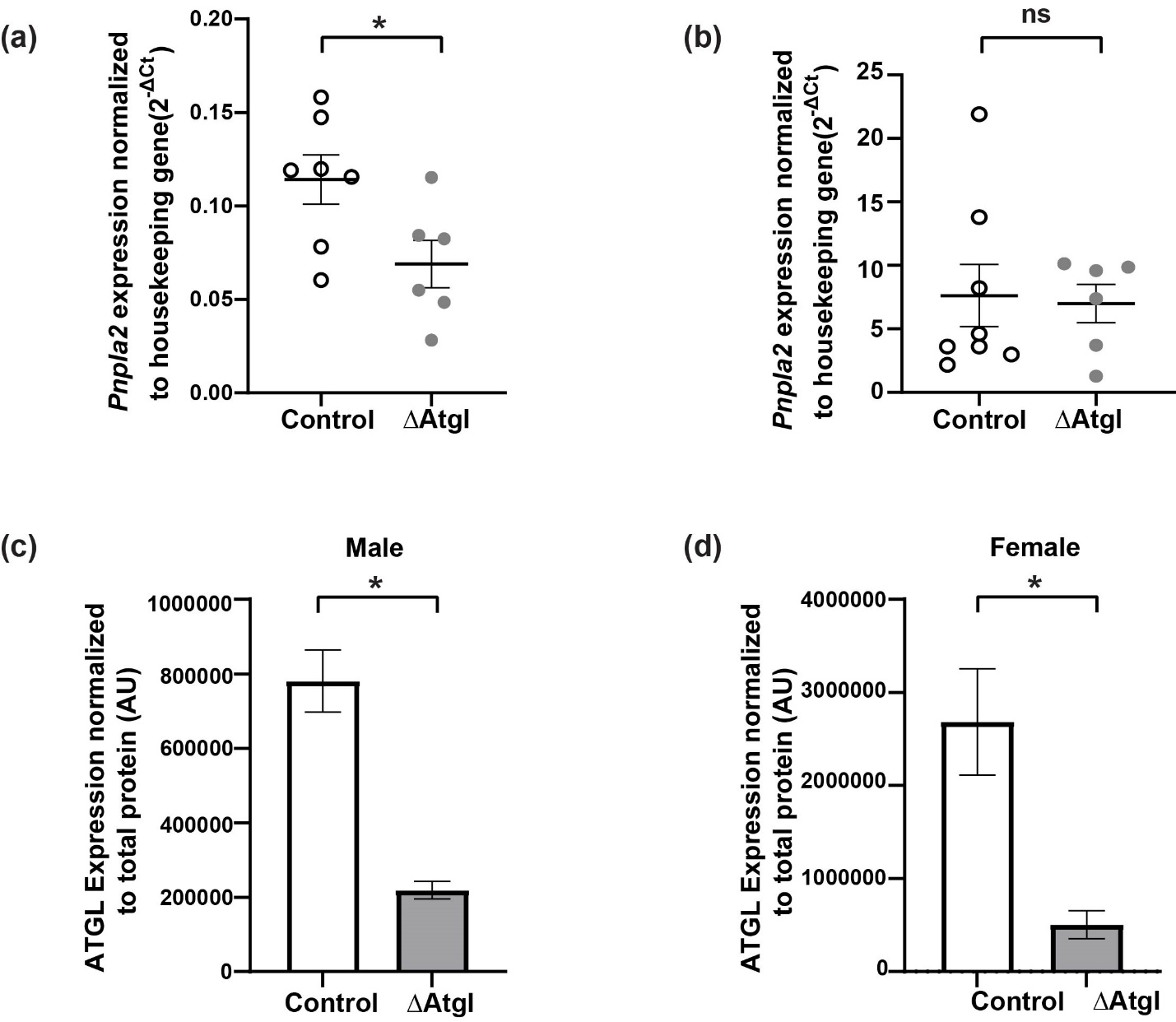


**Figure S7. Expression of ATGL in ΔATGL mice.** Quantitative real time PCR derived normalized expression of *Pnpla2* in (a) flushed femur and (b) epididymal white adipose tissue (eWAT) of 12-week-old male control (open circle) and ΔATGL mice (closed gray circle). Each dot represents data from individual animal where *n*=7 in control and 6 in knock out and data are mean ± standard error of mean. t tests or non-parametric Mann-Whitney tests were done accordingly after testing normal distribution using Shapiro-Wilk normality test to determine significance between two groups where *, p <0.05, **, p <0.01, ***, p <0.001, ****, p <0.0001. Quantification of ATGL expression in femur cortex of (c) male and (d) female control (open bar) and ΔATGL (gray bar) mice normalized to total protein in respectivel lanes measured by no-stain protein labelling. Data are means ± standard error of mean from N=2 for male and N=3 for female mice respectively. Paired T-tests or non-parametric Mann-Whitney tests were done accordingly after testing normal distribution using Shapiro-Wilk normality test to determine significance between two groups where *, p <0.05, **, p <0.01, ***, p <0.001, ****, p <0.0001.


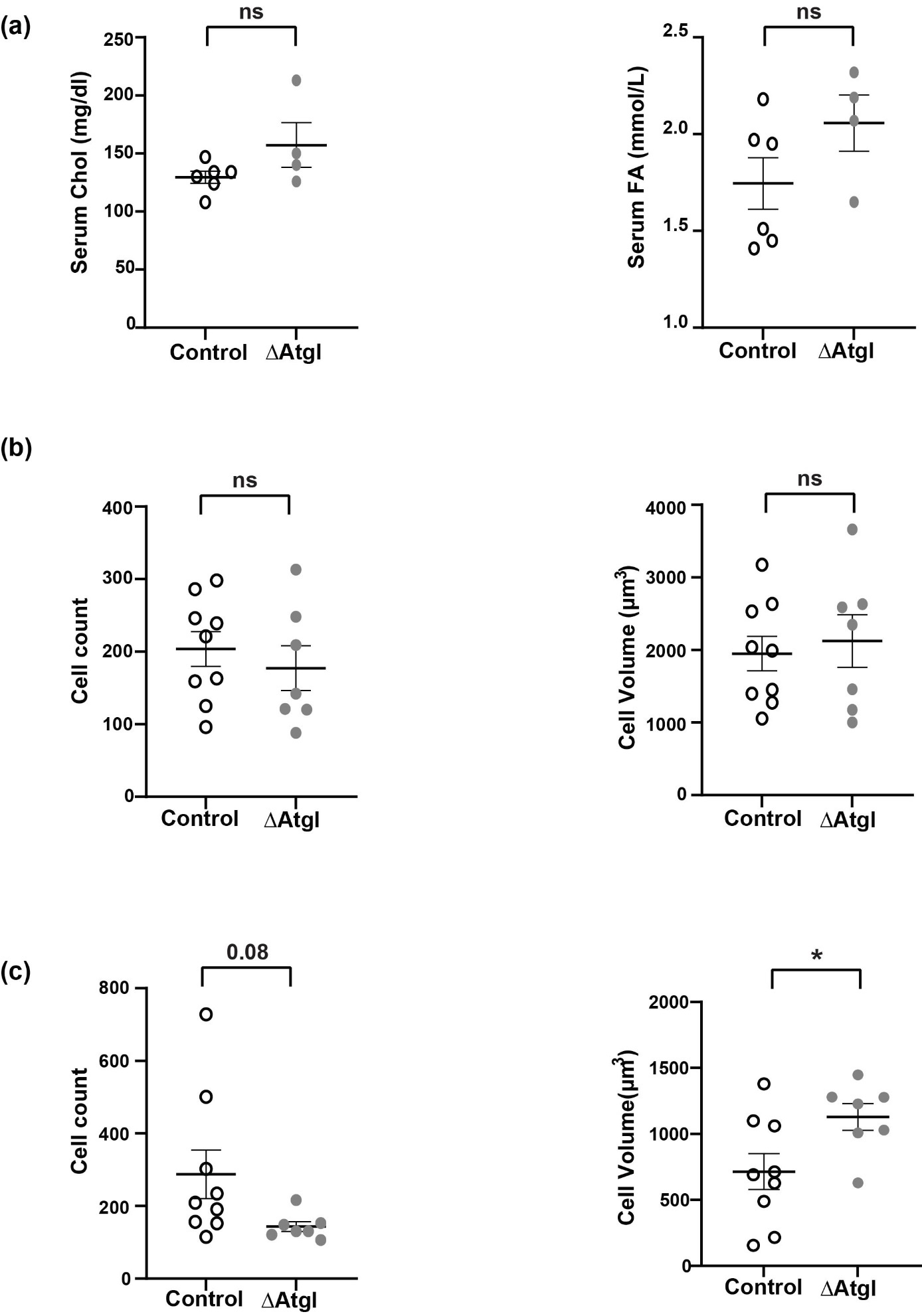


**Figure S8. Serum Lipid and Adipose tissue profile (a)** Serum cholesterol (Chol) and fatty acid (FA) level of 12-week-old control (open circle) and ΔATGL (closed gray circle) mice. (b) Epidedymal, (c) Subcutaneous adipocyte number and volume measured by histological analysis of H&E-stained adipocyte tissue section from 12-week-old control (open circle) and ΔATGL (closed gray circle) mice. Each dot represents data from individual animal where *n*=6 in control and 4 in knock out and data are mean ± standard error of mean. t-tests or non-parametric Mann-Whitney tests were done accordingly after testing normal distribution using Shapiro-Wilk normality test to determine significance between two groups where *, p <0.05, **, p <0.01, ***, p <0.001, ****, p <0.0001.


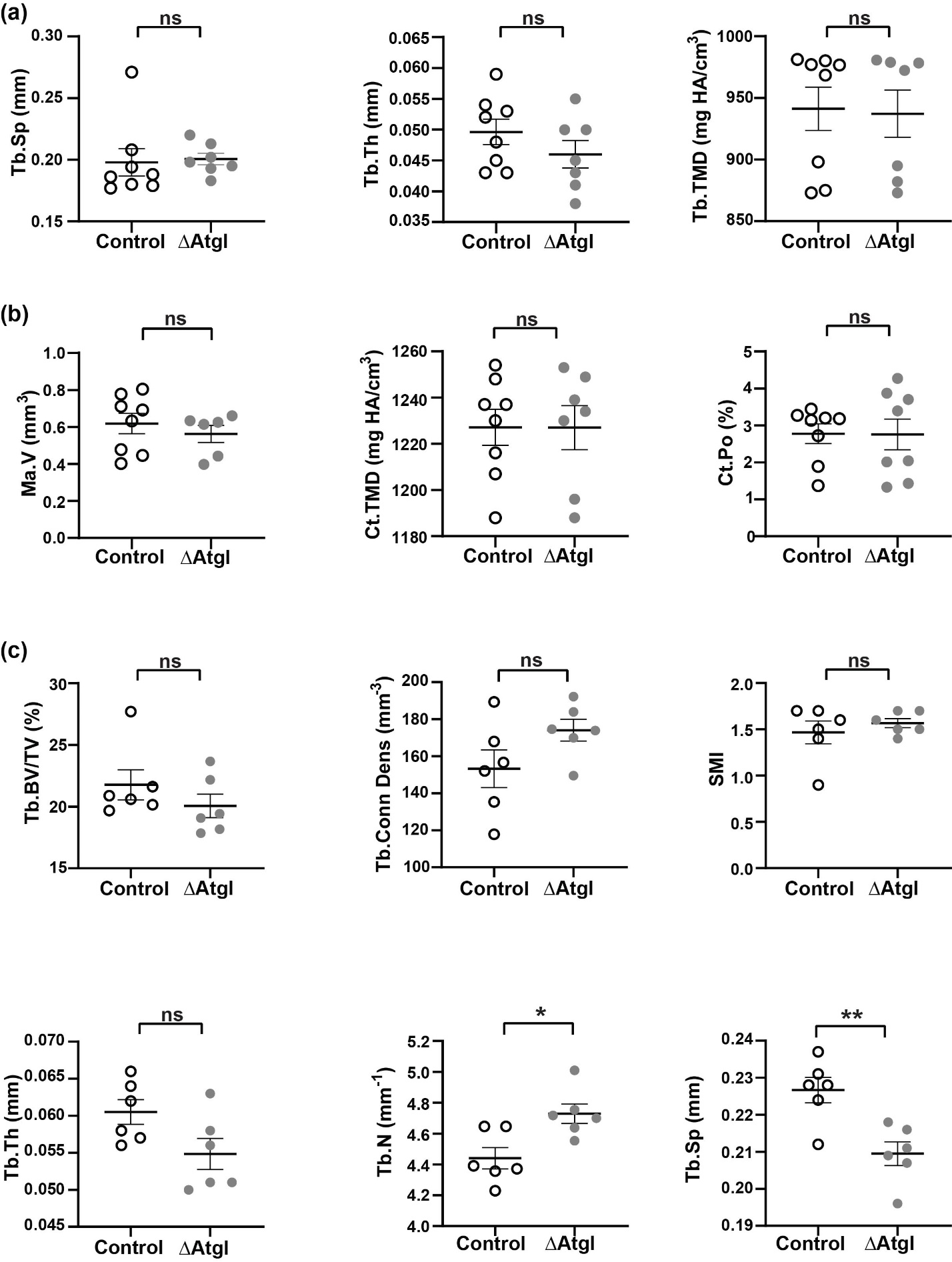


**Figure S9. Micro CT Parameters for ΔATGL male mice.** Micro computed tomography (µCT) analysis of (a) trabecular and (b) cortical bone of tibia of 12-week-old control (open circle) and ΔATGL mice (closed gray circle). Each dot represents data from individual animal where *n*=8 in control and 7 in knock out ΔATGL. Data are mean ± standard error of mean. Paired T-tests or non-parametric Mann-Whitney tests were done accordingly after testing normal distribution using Shapiro-Wilk normality test to determine significance between two groups where *, p <0.05, **, p <0.01, ***, p <0.001, ****, p <0.0001. (c) Micro computed tomography (µCT) analysis of trabecular L5 vertebra of 12-week-old control (open circle) and ΔATGL male mice (closed gray circle). Each dot represents data from individual animal where *n*=6 in both control and knock out and data are mean ± standard error of mean. T-tests or non-parametric Mann-Whitney tests were done accordingly after testing normal distribution using Shapiro-Wilk normality test to determine significance between two groups where *, p <0.05, **, p <0.01, ***, p <0.001, ****, p <0.0001.


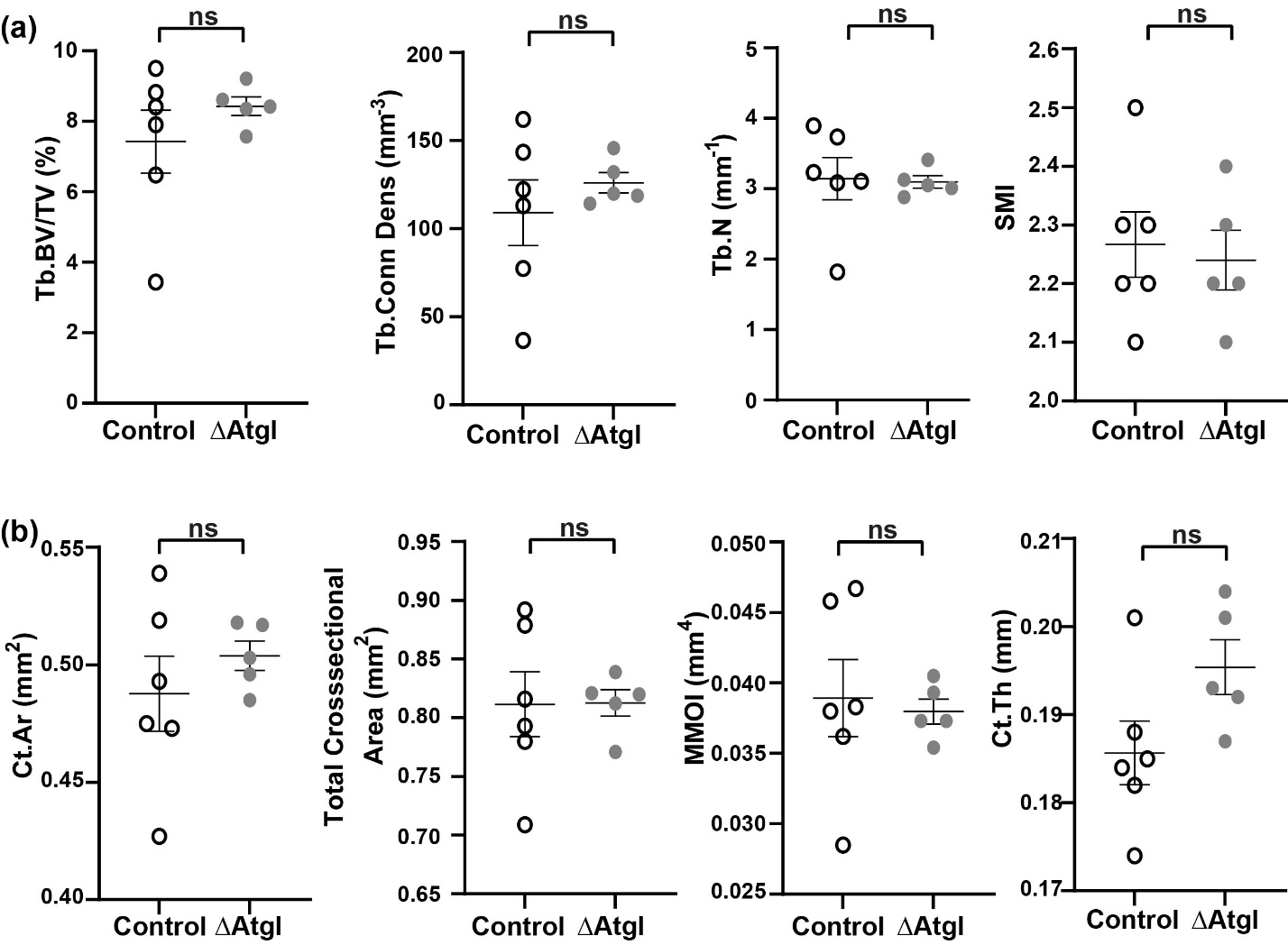


**Figure S10. MicroCT Analysis of Tibia Microarchitecture of Female Mice.** Micro computed tomography (µCT) analysis of (a) trabecular and (b) cortical bone of tibia of 12-week-old control (open circle) and ΔATGL female mice (closed gray circle). Each dot represents data from individual animal where *n*=6 in control and 5 in knock out. Data are mean ± standard error of mean. Paired T-tests or non-parametric Mann-Whitney tests were done accordingly after testing normal distribution using Shapiro-Wilk normality test to determine significance between two groups where *, p <0.05, **, p <0.01, ***, p <0.001, ****, p <0.0001.


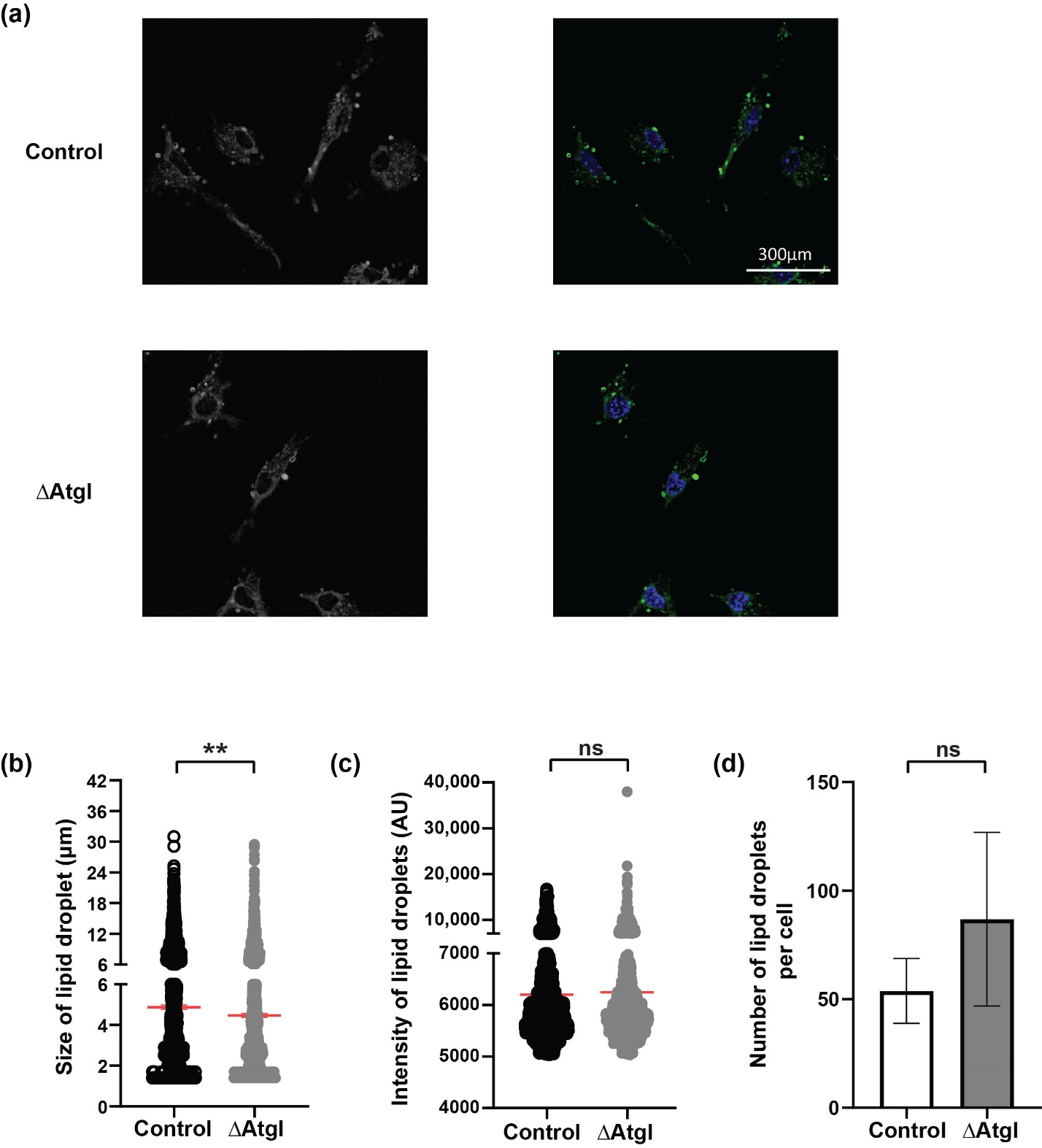


**Figure S11. Lipid metabolism in stromal cells of ΔATGL mice.** (a) Representative confocal image of stromal cells on 0^th^ day before differentiation from control and ΔATGL mice where cellular lipid droplets were stained with BODIPY 493/503 (green in merged panel) along with mounting with DAPI (blue in merged panel) in presence or absence of ATGListatin. Panel 1 show monochrome images of lipid droplet whereas panel 2 shows merged image. Quantification of (b) size, (c) intensity of BODIPY 493/503 stained lipid droplet in differentiated osteoblasts from control (open circle) or in ΔATGL mice (gray closed circle) where each dot represents size or intensity of one lipid droplet. Data are mean ± standard error of mean where size or intensity of each lipid droplets were measured from independent images captured in 6 different field of view of the coverslip (n=6) from pooled BMSCs obtained from 12-week-old mice of each group (N=6 control and N=4 knock out). t tests were done assuming normal distribution since, data points were more than 40, to determine significance between two groups where *, p <0.05, **, p <0.01, ***, p <0.001, ****, p <0.0001. (D) Quantification of number of lipid droplets per cell in differentiated osteoblasts from control (open) and ΔATGL mice (gray) Data and are mean ± standard deviation (SD) where lipid droplets per cell were counted from independent images captured in 6 different field of view (*n*=6) from pooled BMSCs obtained from 12-week-old mice of each group (*n*=6 control and *n*=4 knock out). Number of lipid droplets counted from each image were divided by number of cells (number of DAPI positive nucleus) in that image to get lipid droplets per cell. t tests or non-parametric Mann-Whitney tests were done accordingly after testing normal distribution using Shapiro-Wilk normality test to determine significance between two groups where *, p <0.05, **, p <0.01, ***, p <0.001, ****, p <0.0001.
